# A stably self-renewing adult blood-derived induced neural stem cell exhibiting patternability and epigenetic rejuvenation

**DOI:** 10.1038/s41467-018-06398-5

**Published:** 2018-10-02

**Authors:** Chao Sheng, Johannes Jungverdorben, Hendrik Wiethoff, Qiong Lin, Lea J. Flitsch, Daniela Eckert, Matthias Hebisch, Julia Fischer, Jaideep Kesavan, Beatrice Weykopf, Linda Schneider, Dominik Holtkamp, Heinz Beck, Andreas Till, Ullrich Wüllner, Michael J. Ziller, Wolfgang Wagner, Michael Peitz, Oliver Brüstle

**Affiliations:** 1 Institute of Reconstructive Neurobiology, Life & Brain Center, University of Bonn Medical Center, 53127 Bonn, Germany; 2 German Center for Neurodegenerative Diseases (DZNE), 53175 Bonn, Germany; 3 Helmholtz-Institute for Biomedical Engineering, RWTH Aachen University Medical School, 52074 Aachen, Germany; 4 Institute of Experimental Epileptology and Cognition Research, Life & Brain Center, University of Bonn Medical Center, 53127 Bonn, Germany; 5 Department of Neurology, University of Bonn Medical Center, 53105 Bonn, Germany; 6 Max Planck Institute of Psychiatry, 80804 Munich, Germany

## Abstract

Recent reports suggest that induced neurons (iNs), but not induced pluripotent stem cell (iPSC)-derived neurons, largely preserve age-associated traits. Here, we report on the extent of preserved epigenetic and transcriptional aging signatures in directly converted induced neural stem cells (iNSCs). Employing restricted and integration-free expression of SOX2 and c-MYC, we generated a fully functional, bona fide NSC population from adult blood cells that remains highly responsive to regional patterning cues. Upon conversion, low passage iNSCs display a profound loss of age-related DNA methylation signatures, which further erode across extended passaging, thereby approximating the DNA methylation age of isogenic iPSC-derived neural precursors. This epigenetic rejuvenation is accompanied by a lack of age-associated transcriptional signatures and absence of cellular aging hallmarks. We find iNSCs to be competent for modeling pathological protein aggregation and for neurotransplantation, depicting blood-to-NSC conversion as a rapid alternative route for both disease modeling and neuroregeneration.

## Abstract (web summary)

Induced neurons, but not induced pluripotent stem cell (iPSC)-derived neurons, preserve age-related traits. Here, the authors demonstrate that blood-derived induced neural stem cells (iNSCs), despite lacking a pluripotency transit, lose age-related signatures.

## Introduction

Following the pioneering generation of induced pluripotent stem cells (iPSCs)^1^, numerous studies have corroborated the notion that forced expression of OCT4 alone or together with other pluripotency transcription factors (TFs) is sufficient to induce pluripotency in various somatic cell populations^2–4^. Together with the large repertoire of protocols for controlled differentiation of iPSCs into various tissue-specific cell types, this technology has since enabled patient-specific disease modeling and regeneration for numerous tissues^5,6^. However, in many cases, generation of defined somatic cell types requires complex and lengthy differentiation protocols, which essentially recapitulate embryonic development in vitro^6,7^. At the same time, the concept of TF-based reprogramming has provided the ground for exploring more direct routes for fate conversion of somatic cells. Forced expression of neurogenic TFs suffices to convert mouse and human fibroblasts directly into induced neurons (iNs)^8–10^. An inherent shortcoming of iNs is the fact that the resulting neurons are postmitotic, which precludes further expansion and thus the production of quality-controlled batches. In addition, only a fraction of the fibroblasts undergoes successful neuronal conversion. Emerging evidence further indicates that iNs, in contrast to embryonic stem cell (ESC)- and iPSC-derived neurons, largely retain age-associated transcriptomic and epigenetic signatures^11,12^. These properties might serve as an asset for modeling age-related disorders, but at the same time pose severe limitations for therapeutic applications.

More recently, several studies have addressed the direct conversion of human somatic cells into induced neural precursor cells (iNPCs)^13–18^. However, most of the initial protocols still employed the pluripotency factor OCT4, which has been discussed to induce a transient state of pluripotency instead of a genuine direct cell fate conversion process^19,20^. Furthermore, neural cells generated with pluripotency factors such as OCT4 were found to display significantly more genomic aberrations and less chromosomal stability compared to iNs and iNPCs generated using only neural lineage-specific TFs^21^. While recent studies reported on OCT4-free protocols for direct conversion of neonatal human tissues such as umbilical cord blood and foreskin fibroblasts into expandable iNPCs, the generation of adult human tissue-derived early-stage NSCs featuring long-term self-renewal, clonogenicity, tripotency, and responsiveness to lineage patterning cues remains a challenge^13,16,18,22^.

Here, we set out to devise a protocol for direct, efficient, and OCT4-free generation of bona fide iNSCs. To facilitate the derivation process we used adult human peripheral blood cells (PBCs) instead of skin fibroblasts, which come with the requirement of an invasive surgical procedure, increased risk of genetic aberrations due to environmental exposure, and a lengthy expansion process with the risk of introducing de novo mutations. We show that iNSCs generated with non-integrating vectors under defined conditions are capable of self-renewal and tripotent differentiation at the single cell level, and remain responsive to instructive patterning and differentiation cues promoting specification of neuronal and glial subtypes. Most importantly, we demonstrate that age-associated DNA methylation (DNAm) patterns are largely erased in our iNSCs when compared to neural precursor cells (NPCs) derived from isogenic iPSCs. Furthermore, we found that iNSCs generated via our OCT4-free approach lack age-associated transcriptional signatures and other cellular aging hallmarks. Finally, we provide proof-of-principle data supporting the applicability of iNSCs for modeling neurodegenerative diseases and for neural transplantation.

## Results

### Direct conversion of adult human PBCs into iNSCs

**Fig. 1:**
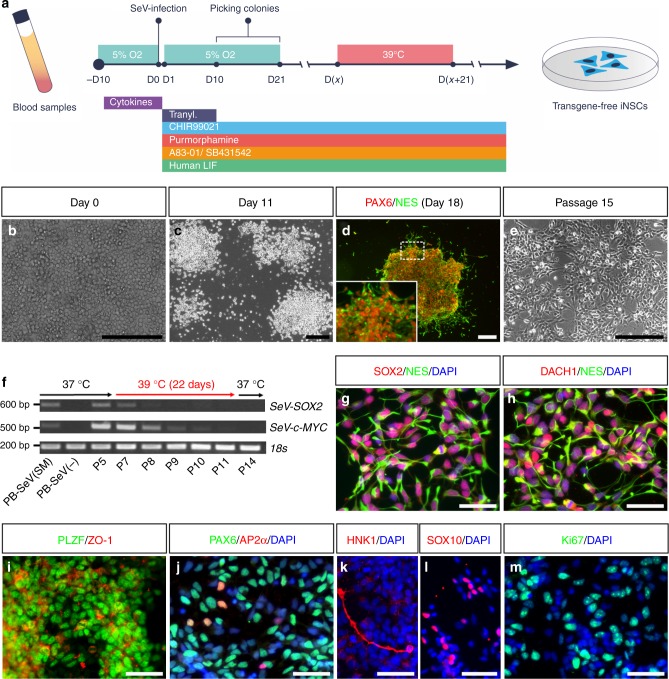
Direct conversion of adult human PBCs into iNSCs. **a** Schematic representation of PB-iNSC generation. **b**–**e** Within 1 week after infection of PBCs (**b**), actively proliferating neuroepithelial colonies (**c**) immunopositive for early NSC markers PAX6 and NES (**d**) emerge, which can be further expanded to form stably self-renewing iNSCs (**e**). **f** RT-PCR confirms the fast decline of *SeV-SOX2* and *SeV-c-MYC* expression during 39 °C incubation and the persistent absence of SeV genomes in iNSC cultures. **g**–**l** Transgene-free iNSCs express typical neural lineage markers, including early neuroectoderm and neural rosette markers SOX2 (**g**), NES (**g**, **h**), DACH1 (**h**), PLZF (**i**), ZO-1 (**i**), and PAX6 (**j**). A small fraction of cells is immunopositive for the neural crest markers AP2α (**j**, 2.0 ± 0.7%, mean ± s.d., *n* = 3), HNK1 (**k**, 17.5 ± 8.3%, *n* = 3), and SOX10 (**l**, 2.4 ± 0.3%, *n* = 3). **m** Proliferating iNSCs show prominent expression of the proliferation marker Ki67. P5–P14: passage numbers; PB-SeV (SM): PBCs infected with SeV-SOX2 and SeV-c-MYC; PB-SeV (−): PBCs without infection. Scale bars: 200 μm (**b**–**e**); 50 μm (**g**–**m**)

To address the question whether adult PBCs can be directly converted into stably expandable multipotent iNSCs (PB-iNSCs), we collected 6 peripheral blood (PB) samples from donors at different ages (31–62 years) and derived erythroblasts^23^ as starting cells (Fig. 1a, b). After infection with non-integrating Sendai viruses (SeV) expressing SOX2 and c-MYC, we found that a medium condition supplemented with the GSK3β inhibitor CHIR99021 (CHIR), the Hedgehog activator purmorphamine, the ALK-5 inhibitor A83-01, recombinant human LIF (hLIF), and tranylcypromine (Tranyl) together with a hypoxic atmosphere enables fast and efficient neural cell fate conversion. Within 1 week, transduced cells attached, elongated, and formed colonies of neuroepithelial cells, which can be picked manually starting from day 10 until day 21 after transduction (Fig. 1c). Neuroepithelial colonies expressing early NSC markers such as NES and PAX6 (Fig. 1d) were mechanically triturated into small clusters, re-plated in matrigel-coated dishes in iNSC expansion medium supplemented with CHIR, purmorphamine, A83-01, and hLIF, and cultured under normoxic conditions thereafter (Fig. 1e). Stably expandable, multipotent iNSCs could be generated from all 6 PBC samples with efficiencies ranging from 0.08 to 0.66%. This is comparable to a previous study, in which the efficiency for converting human neonatal foreskin fibroblasts into iNSCs was reported to be around 0.4%^22^. To monitor the transition from a PBC into a NSC fate, we assessed the expression of characteristic erythroblast-, neuroectoderm-, and pluripotency-associated genes at days 0, 3, 7, and 14 after infection (Supplementary Fig. 1), and detected a fast up-regulation of the neuroectodermal markers *SOX2*, *PAX6*, *NES*, and *CDH2* with concomitant down-regulation of the erythroblast markers *CD71* and *CD117* within the first 2 weeks of conversion. At no time point did we detect expression of the key pluripotency gene *OCT4* and the reprogramming-associated gene *SALL4*. These data indicate a rapid and robust blood-to-NSC conversion process without the formation of an intermediate iPSC-like state.

Elimination of the temperature-sensitive SeV vectors was facilitated by cultivation at 39 °C for about 3–4 weeks. PCR analyses uncovered a fast decline of exogenous *SOX2* expression, whereas *SeV-c-MYC* expression was more resistant to the high-temperature treatment; a phenomenon described in other studies^24^. However, both transgenes were no longer detectable after about 3–4 weeks of cultivation and remained absent thereafter (Fig. 1f and Supplementary Fig. 2). Immunostaining revealed that transgene-free PB-iNSCs express a wide range of early neuroectoderm and neural rosette markers, such as SOX2, NES, DACH1, PLZF, ZO-1, and PAX6 (Fig. 1g–j). Interestingly, a small fraction of cells was immunopositive for neural crest markers, such as AP2α (2.0 ± 0.7%, mean ± s.d.; *n* = 3), HNK1 (17.5 ± 8.3%, *n* = 3), and SOX10 (2.4 ± 0.3%, *n* = 3) (Fig. 1j–l), suggesting an early developmental stage of PB-iNSCs with the potential to still give rise to derivatives of both central and peripheral nervous system (CNS and PNS). PB-iNSCs showed prominent expression of the proliferation marker Ki67 (Fig. 1m), indicating strong self-renewal capacity. Indeed, transgene-free iNSCs generated on matrigel or mouse embryonic fibroblasts (MEFs) could be stably expanded for more than 20 passages while maintaining a normal karyotype (Supplementary Fig. 3). In contrast to isogenic iPSCs and iPSC-derived small molecule NPCs (smNPCs)^25^, telomeres of iNSCs were not elongated during the direct conversion process (Supplementary Fig. 4), which is in line with the notion that these cells do not traverse an intermediate iPSC-like state.

**Fig. 2:**
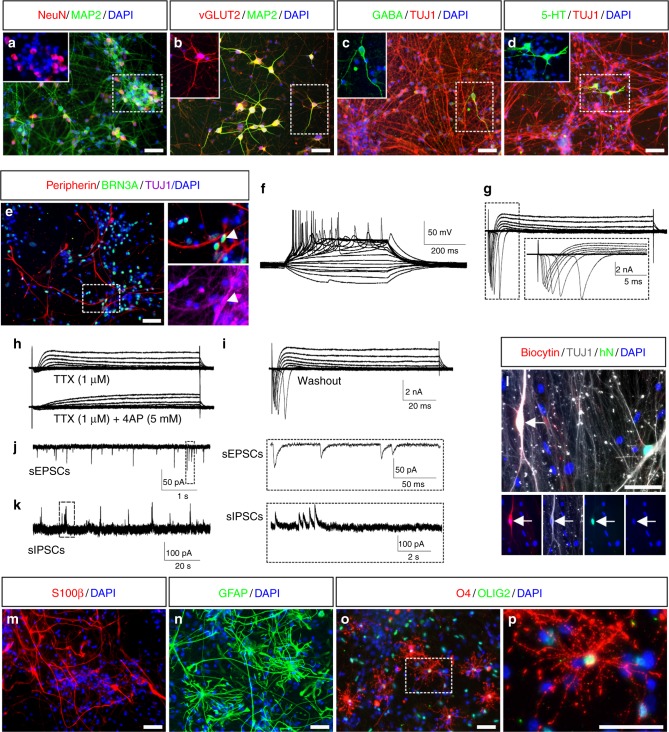
iNSCs give rise to functional neurons, astrocytes, and oligodendrocytes. **a** Six weeks after differentiation, iNSC-derived neurons express markers of mature neurons such as MAP2 and NeuN. **b**–**d** Upon spontaneous differentiation, iNSCs give rise to vGLUT2-positive glutamatergic neurons (**b**). In addition, staining for GABA (**c**) or serotonin (5-HT, **d**) can be detected. **e** Peripherin^+^/BRN3A^+^/TUJ1^+^ PNS neurons in 30-day-differentiated iNSCs. Arrowhead highlights an example of a triple-positive cell. **f**–**l** Whole-cell current clamp measurements reveal the generation of multiple action potentials upon depolarizing current injections (**f**, *n* = 6/7). In response to depolarizing voltage steps, these neurons elicit fast and transient TTX-sensitive sodium-dependent inward currents and sustained 4-AP-sensitive potassium-dependent outward currents (**g**–**i**, *n* = 6/6). Spontaneous postsynaptic currents are observed in whole-cell voltage-clamp recordings, indicating the in vitro formation of functional excitatory (**j**, *n* = 6/7) and inhibitory (**k**, *n* = 1/8) synapses. Post-hoc immunostaining reveals co-localization of biocytin, TUJ1, and the human-specific marker hN in the recorded neurons (arrows, **l**). **m**–**p** iNSCs give rise to S100β^+^ (**m**) and GFAP^+^ (**n**) astrocytes as well as O4^+^ and OLIG2^+^ (**o**, **p**) oligodendrocytes. Scale bars: 50 μm

Next, we assessed the differentiation potential of PB-iNSCs upon growth factor withdrawal. TUJ1^+^ neurons appeared within 3 weeks, and markers for more mature neurons such as MAP2 and NeuN were readily detectable after 6 weeks of spontaneous differentiation (Fig. 2a). iNSC-derived neurons strongly expressed vGLUT2, a marker of glutamatergic neurons (Fig. 2b). Differentiated cultures also contained neurons immunopositive for γ-aminobutyric acid (GABA) or 5-hydroxytryptamine (5-HT), indicating the presence of GABAergic and serotonergic neurons, respectively (Fig. 2c, d). In addition to CNS neurons, PB-iNSCs spontaneously generated TUJ1^+^ neurons co-expressing BRN3A and peripherin, markers typically expressed in PNS neurons (Fig. 2e). These results strongly indicate that PB-iNSCs can give rise to multiple neuronal subtypes of both CNS and PNS.

To study the electrophysiological properties of iNSC-derived neurons, patch-clamp recordings were performed on neurons differentiated for 8–12 weeks on primary mouse astrocytes^10^ (Fig. 2f–l). Whole-cell current clamp measurements revealed the generation of multiple action potentials in 6 out of 7 neurons upon depolarizing current injections (Fig. 2f). In response to depolarizing voltage steps, 6 out of 6 iNSC-derived neurons generated fast and transient sodium-dependent inward currents and sustained potassium-dependent outward currents (Fig. 2g). Sodium and potassium currents could be blocked by Tetrodotoxin (TTX) and 4-Aminopyridine (4-AP), and recovered after washing out the drugs (Fig. 2h, i). Spontaneous excitatory postsynaptic currents (sEPSCs) were observed in 6 out of 7 neurons, and spontaneous inhibitory postsynaptic currents (sIPSCs) were detected in 1 out of 8 neurons in whole-cell voltage-clamp recordings, indicating excitatory and inhibitory functional synapse formation in vitro (Fig. 2j, k). Post-hoc immunostaining of neurons, which were injected with biocytin during recording, revealed co-localization of biocytin, TUJ1, and human nuclei antigen (hN), confirming their human identity (Fig. 2l). PB-iNSCs also spontaneously generated S100β^+^ and GFAP^+^ astrocytes (Fig. 2m, n). Applying a recently established 3-stage-protocol for oligodendroglial differentiation^26^, we were further able to differentiate PB-iNSCs efficiently into oligodendrocytes (Fig. 2o, p and Supplementary Fig. 5).

We next addressed the question whether our transgene-free PB-iNSC cultures contain bona fide stem cells capable of self-renewal and tripotent differentiation at the single cell level. To that end, single iNSCs were deposited in matrigel-coated 96-wells using fluorescence-activated cell sorting (FACS). Out of 13 wells containing single cells, 8 proliferated to form subclones exhibiting typical neuroepithelial morphologies (Supplementary Fig. 6a). Subcloned iNSC lines could be proliferated across at least 12 passages while maintaining the expression of CNS and PNS lineage markers (Supplementary Fig. 6b). Three iNSC subclones were assessed for their differentiation capacity, and all three of them were able to generate neurons, astrocytes, and oligodendrocytes (Supplementary Fig. 6c). From these observations, we conclude that PB-iNSCs represent bona fide neural stem cells that are stably expandable and tripotent at the single cell level.

### iNSCs respond to regional patterning cues

**Fig. 3:**
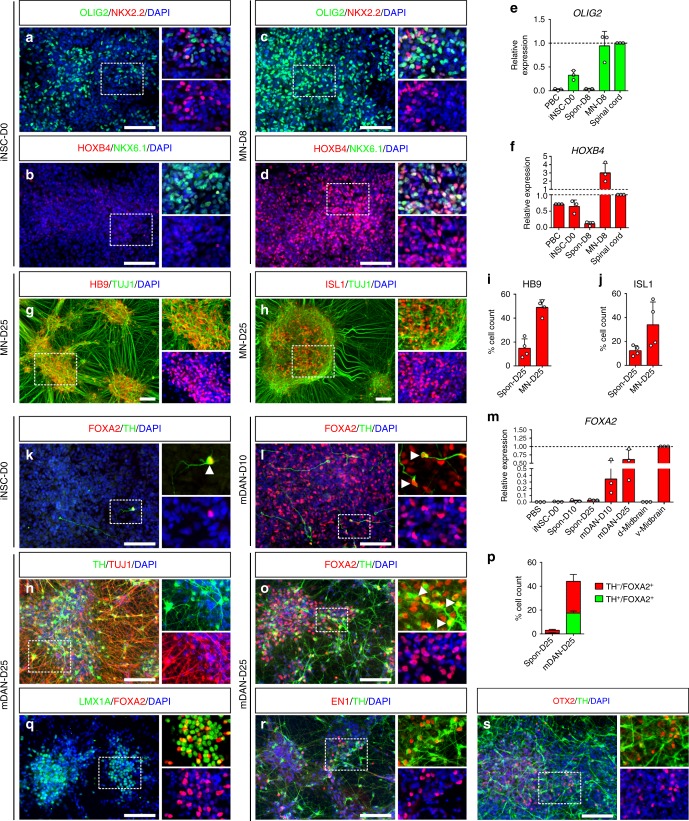
Directed differentiation of iNSCs into motoneurons and midbrain dopamine-like neurons. **a**–**f** Activation of SHH and retinoic acid signaling enables efficient specification of iNSCs (**a**, **b**) into OLIG2^+^ and HOXB4^+^ motoneuron progenitors (**c**, **d**), while the expression of other ventral markers such as NKX2.2 or NKX6.1 remains unchanged (**a**–**d**). qPCR confirms the upregulation of *OLIG2* (**e**) and *HOXB4* (**f**) in iNSCs upon the treatment with patterning factors (*n* = 3). **g**–**j** Patterned iNSCs further differentiate into a highly enriched HB9^+^/TUJ1^+^ (**g**) and ISL1^+^/TUJ1^+^ (**h**) motoneuron population. Quantification of HB9^+^ (**i**) and ISL1^+^ (**j**) cells at day 25 confirms an enriched motoneuron fraction induced by patterning compared to spontaneous differentiation (*n* = 4). **k**–**m** Inhibition of GSK3β signaling and activation of SHH signaling robustly induce the expression of FOXA2 in iNSCs (**k**, **l**). qPCR confirms the upregulation of *FOXA2* in iNSCs upon the application of patterning factors (**m**, *n* = 3). **n**–**p** Patterned iNSCs efficiently give rise to TH^+^/TUJ1^+^ dopamine neurons (**n**). Quantification of FOXA2^+^ and FOXA2^+^/TH^+^ cells at day 25 confirms an enriched midbrain dopamine-like neuronal population induced by patterning compared to spontaneous differentiation (**o**, **p**, *n* = 3). Arrowheads highlight examples of FOXA2^+^/TH^+^ cells. **q**–**s** Cells express midbrain dopamine neuron markers LMX1A (**q**), EN1 (**r**), and OTX2 (**s**) at day 25 of the midbrain patterning protocol. Single fluorescence channels are shown in the lower right close-ups of each panel. Data are presented as mean + s.d.; *n* = 3–4. iNSC-D0: undifferentiated iNSCs at day 0; MN-D8, D25: day 8, day 25 of motoneuron differentiation; mDAN-D10, D25: day 10, day 25 of midbrain dopamine-like neuron differentiation; Spon-D8, D10, D25: day 8, day 10, day 25 of spontaneous differentiation; v-Midbrain: ventral midbrain; d-Midbrain: dorsal midbrain. Scale bars: 100 μm

Previous studies have shown that pluripotent stem cell (PSC)-derived NSCs can exhibit distinct regional identities, which might be due to growth factors and morphogens used during neural induction and/or subsequent expansion^26–28^. Our PB-iNSCs were converted and expanded in conditions containing CHIR and purmorphamine, which promote the formation of posterior and ventral fates, respectively. Indeed, ventral and posterior brain markers such as *OLIG2*, *NKX2.2*, *NKX6.1*, and *HOX* genes were readily detectable in PB-iNSCs at RNA and/or protein levels (Fig. 3a, b and Supplementary Fig. 7a), whereas dorsal and anterior markers such as *PAX3* and *FOXG1* were absent in long-term iNSC cultures (Supplementary Fig. 7a). These data suggest a posterior ventral identity of PB-iNSCs. In line with this, iNSCs spontaneously gave rise to neurons expressing LIM1/2 and LIM3, markers typically found in ventral hindbrain interneurons, as well as the motoneuron markers HB9 and ISL1 (Supplementary Fig. 7b, c). While we also detected TH^+^ neurons, FOXA2, a key marker for floor-plate and midbrain dopamine neurons, was rarely observed (Supplementary Fig. 7d).

To study whether PB-iNSCs remain responsive to instructive regionalization cues, we applied a small molecule treatment protocol for motoneuron induction (Supplementary Fig. 7e)^29^. After 8 days of differentiation with patterning molecules including retinoic acid, purmorphamine, and CHIR, the cells exhibited a ventral, upper spinal cord identity with increased HOXB4 and OLIG2 expression, markers typically found in motoneuron progenitors (Fig. 3a–d). The upregulation of *OLIG2* and *HOXB4* expression upon patterning treatment was further confirmed by quantitative RT-PCR (qPCR, Fig. 3e, f). The patterning treatment did not affect the expression of other ventral markers such as NKX2.2 or NKX6.1 (Fig. 3a–d). After 25 days of differentiation, the patterned cultures gave rise to a highly enriched HB9^+^ (48.9 ± 6.5%, *n* = 4) and ISL1^+^ (33.9 ± 19.1%, *n* = 4) motoneuron population. In comparison, only 14.8 ± 7.9% HB9^+^ (*n* = 4) and 12.3 ± 4.2% ISL1^+^ (*n* = 4) cells were found in spontaneously differentiated cultures (Fig. 3g–j).

We then sought to explore whether the regional identity of iNSCs could be shifted towards other neuronal lineages and exposed the cells to a set of patterning factors used for the differentiation of PSCs into midbrain dopamine neurons, including CHIR, sonic hedgehog (SHH), and fibroblast growth factor 8 (FGF8; Supplementary Fig. 7f)^30,31^. Within 10 days we observed a striking increase of FOXA2-expressing cells (Fig. 3k, l), which was further confirmed by qPCR analysis (Fig. 3m). After 25 days of differentiation, 46.7 ± 3.5% (*n* = 3) of the cells were positive for FOXA2 with 37.5 ± 1.7% (*n* = 3) of them co-expressing TH (Fig. 3n–p). Fractions of these cells also expressed LMX1A, EN1, and OTX2, midbrain-associated markers typically found in dopamine neurons (Fig. 3q–s). In contrast, untreated control cultures contained only 3.1 ± 0.8% (*n* = 3) FOXA2^+^ cells (Fig. 3p). From these data, we conclude that PB-iNSCs can respond to different instructive patterning and differentiation cues and efficiently give rise to specific neuronal subpopulations such as motoneurons and midbrain dopamine-like neurons. Similar data were obtained using iNSCs generated from neonatal umbilical cord blood (CB-iNSCs), an alternative and easily accessible resource for reprogramming studies (Supplementary Fig. 8).

### Comparative global profiling of iNSCs and PSC-derived NPCs

**Fig. 4:**
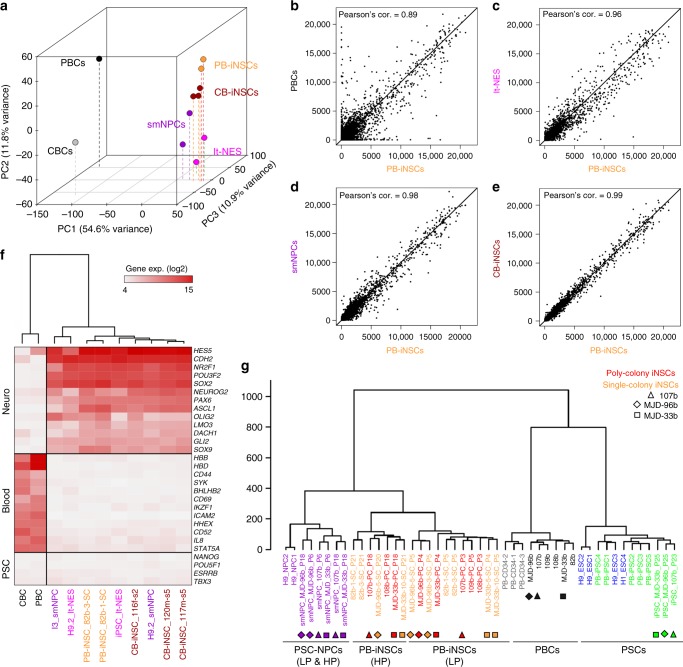
Global gene expression and DNA methylation analysis. **a** Principal component analysis indicates that both PB-iNSCs and CB-iNSCs exhibit global gene expression profiles similar to those of their PSC-derived counterparts, but clearly distinct from their parental blood cells. **b**–**e** Scatter plot analysis of global gene expression profiles shows that PB-iNSCs are very distinct from PBCs (**b**). PB-iNSCs resemble lt-NES (**c**) and display a particularly high similarity to smNPCs (**d**). PB-iNSCs show a high degree of similarity with CB-iNSCs (**e**). Principal component analysis (**a**) and pair-wise scatterplots (**b**–**e**) were done genome-wide including 31,427 genes without filtration. **f** Heatmap of selected genes representative for neural, hematopoietic, and pluripotent cells reveals the expression of genes specific to the neural lineage in iNSCs. The clustering of different cell populations is based on the selected genes shown in the heatmap. **g** Hierarchical clustering of global DNAm profiles (485,512 CpGs) shows that the DNAm profiles of iNSCs are similar to those of PSC-derived NPCs and very distinct from PBCs and PSCs. The *y*-axis represents the height of dendrogram calculated using Euclidean distance and Ward's linkage method. PC: principle component; CBCs: CB-CD34^+^ cells; LP: low passages; HP: high passages. Triangles, diamonds, and squares indicate isogenic backgrounds

To assess to what extent human blood-derived iNSCs resemble PSC-derived neural progenies, we compared the global gene expression and DNAm profiles of iNSCs to those of their PSC-derived counterparts and parental blood cells. Principal component analysis (PCA) showed that iNSCs and human PSC-derived NPCs, including long-term neuroepithelial stem cells (lt-NES cells)^27^ and smNPCs^25^, were closely related and distinct from blood cells (Fig. 4a). Pairwise comparisons of gene expression profiles supported the notion that PB-iNSCs differ markedly from PBCs (Fig. 4b, Pearson’s correlation = 0.89). However, iNSCs resembled lt-NES cells (Fig. 4c, Pearson’s correlation = 0.96) and showed particularly high similarity to smNPCs (Fig. 4d, Pearson’s correlation = 0.98). PB-iNSCs displayed a high degree of similarity with CB-iNSCs (Fig. 4e, Pearson’s correlation = 0.99). A heatmap with selected marker genes for neural cells, blood cells, and PSCs revealed that (i) genes playing significant roles in the neural lineage such as *HES5*, *SOX2*, *PAX6*, *ASCL1*, and *DACH1* were strongly up-regulated in iNSCs, reaching levels similar to those observed in PSC-derived NPCs; (ii) genes related to physiological functions of blood cells such as oxygen transport (*HBB*, *HBD*), lymphocyte differentiation/activation (*SYK*, *IKZF1*, *STAT5A*), and wound healing (*CD44*) were down-regulated in iNSCs; (iii) expression of pluripotency genes was absent in all cell populations (Fig. 4f).

We next analyzed the global DNAm profiles of different cell preparations. To this end, we established iNSC lines from either single or multiple neuroepithelial colonies (single-colony or poly-colony iNSCs) from 6 individuals aged 31–62 years (Supplementary Fig. 9 and Supplementary Table 1). In addition, we generated isogenic iPSCs and iPSC-derived smNPCs from 3 of the blood donors (Supplementary Fig. 10 and Supplementary Table 2). We also included publicly available data sets generated from hESCs, hESC-derived NPCs as well as PB-CD34^+^ cells and iPSCs derived from them (PB-iPSCs) (Supplementary Table 2)^32,33^. Global DNA methylation profiling on 485,512 CpG sets revealed that the cell populations clustered into three groups, i.e., PSCs, PBCs, and NPCs including both ESC- and iPSC-derived NPCs and blood-derived iNSCs at low and high passages (Fig. 4g). Within the neural cluster, single- and poly-colony iNSCs shared highly similar DNAm profiles. Interestingly, iNSCs segregated according to low and high passage numbers, suggesting changes of DNAm profiles in iNSC cultures during extended in vitro culture. Thus, despite subtle differences, human blood-derived iNSCs display global gene expression and DNAm profiles which are similar to those of PSC-derived NPCs and markedly distinct from PSCs and blood cells.

### Loss of age-related epigenetic marks in iNSCs

**Fig. 5:**
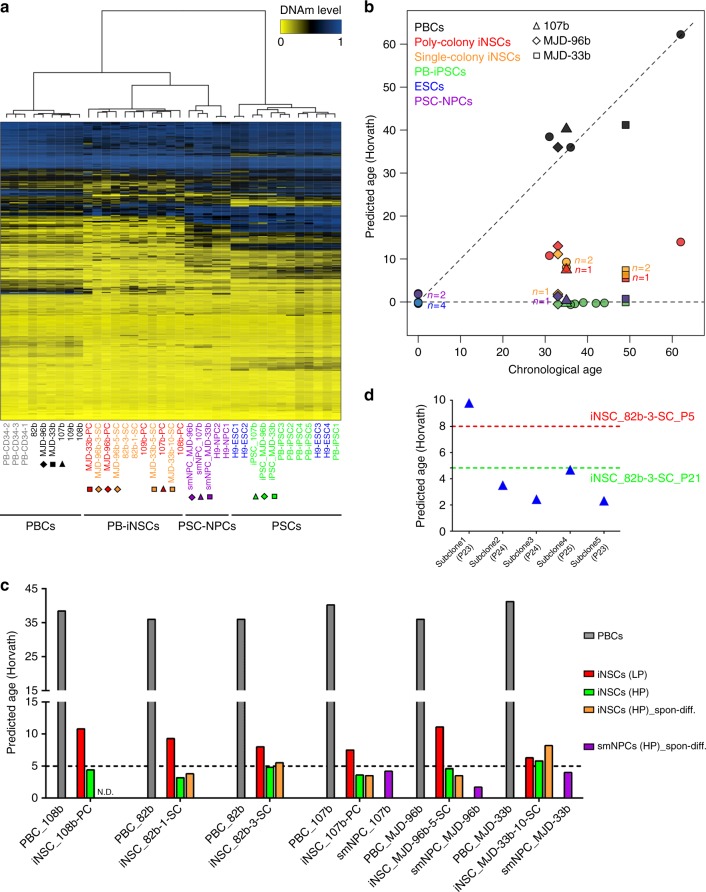
Age-related epigenetic signatures of iNSCs and PSC-derived NPCs. **a** Heatmap representation of DNAm levels at the 353 age-associated CpG sites derived from the Horvath model. The age-associated DNAm patterns of iNSCs differ from PSCs. **b** Age prediction using the Horvath model. Poly- and single-colony-derived low passage iNSC cultures (*P* ≤ 6) show a pronounced loss of age-related epigenetic signatures. Isogenic PB-iPSCs and iPSC-derived smNPCs display epigenetic age signatures similar to ESCs and ESC-derived NPCs. **c** Upon continuous expansion (*P* ≥ 18), a further decline of predicted DNAm age is observed using the Horvath model in 6 independent iNSC lines. Spontaneously differentiated cultures derived from 5 high passage iNSC lines and 3 isogenic high passage smNPC lines (*P* ≥ 18) show similar DNAm ages. **d** Subclones derived from single cells of a single-colony iNSC line (82b-3-SC) exhibit variable DNAm ages (Horvath model). N.D.: not determined

Considering the results of recent studies, which suggest that age-associated transcriptomic and epigenetic traits are much better conserved in directly converted vs. iPSC-derived neurons^11,12^, we assessed age-associated DNAm patterns. Our studies are based on the notion that chronological age is reflected by specific DNAm patterns referred to as “DNAm age”^34^. To examine whether age-associated DNAm patterns are differently preserved in iPSC reprogramming vs. iNSC generation, we employed three multivariate aging models which are based on DNAm levels at age-associated CpG sites^34–36^. In analogy to previous studies, age-associated DNAm patterns at these CpG sites were erased during iPSC reprogramming^34–37^, whereas newly generated iNSCs revealed unique DNAm patterns at age-associated CpGs. In comparison to their parental PBCs, iNSCs displayed a remarkable epigenetic rejuvenation, though not as much as observed for PSCs and PSC-derived NPCs^37^ (Fig. 5a, b and Supplementary Fig. 11). The Horvath model is based on 353 age-associated CpG sites and—in contrast to the other two models—trained by and applicable to multiple tissues and cell types^34^. For that reason, we mainly focused on the Horvath model, which faithfully predicted the chronological age of PBCs. In contrast, PSCs and PSC-derived NPCs including isogenic iPSC-derived smNPCs were predicted to be of ages very close to zero, thus reflecting embryonic-like age signatures (Fig. 5b and Supplementary Tables 1, 2). Interestingly, PB-iNSCs at low passages displayed largely reduced DNAm ages, which ranged between 5.5 and 39.4% of the chronological age of the donor PBCs. Although these DNAm ages are much younger than the corresponding chronological ages, they also point to a partial preservation of age-related traits upon direct neural conversion of PBCs (Fig. 5b and Supplementary Table 1). Age predictions obtained from the two alternative aging models showed a similar pattern with the predicted ages of the iNSCs being located between those of the parental PBCs and the PSCs and PSC-derived NPCs (Supplementary Fig. 11 and Supplementary Table 1). Interestingly, continuous expansion appeared to result in a further decrease of the DNAm ages of iNSCs, as cells at high passages (passage ≥ 18) were predicted younger than cells at low passages (passage ≤ 6), both in poly- and single-colony-derived populations (Fig. 5c and Supplementary Table 1). We further found that iNSC subclones showed variable DNAm ages, suggesting that during the subcloning process each individual cell preserves age-associated epigenetic information to a different degree (Fig. 5d and Supplementary Table 1). We also assessed DNAm signatures of differentiated high passage iNSCs (passage *P* ≥ 18) and detected similar or slightly increased DNAm ages after 2 months of spontaneous differentiation compared to those of undifferentiated iNSCs at high passages (Fig. 5c and Supplementary Table 1). Interestingly, long-term propagated isogenic iPSC-derived smNPCs (passage = 18) subjected to 2 months of in vitro differentiation, too, showed similar or slightly increased DNAm ages compared to those of undifferentiated smNPCs at high passages (Supplementary Table 2). In the end, differentiated cultures generated from iNSCs and isogenic iPSC-derived smNPCs (passage ≥ 18) exhibited comparable DNAm ages (Fig. 5c and Supplementary Tables 1, 2). Thus, in contrast to the results from iN studies, our data argue against an extensive preservation of age-related epigenetic alterations in established iNSC cultures.

### iNSCs lack age-associated transcriptional signatures

**Fig. 6:**
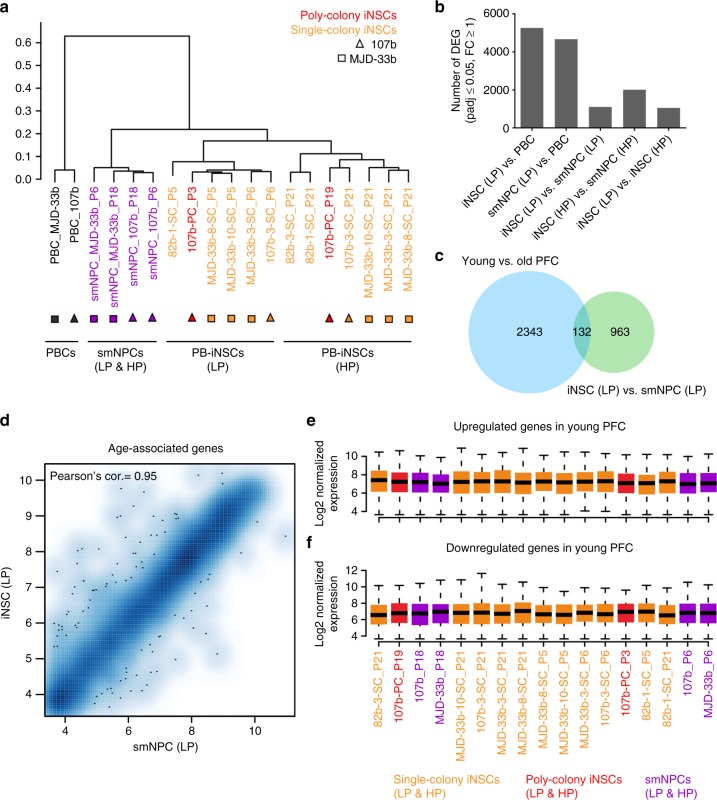
Age-associated transcriptional signatures of iNSCs and PSC-derived NPCs. **a** Clustering of global transcriptional profiles of PBCs, iNSCs, and isogenic smNPCs at low (*P* ≤ 6) and high (*P* ≥ 18) passages. **b** Number of DEGs between different cell populations (*q*-value ≤ 0.05, absolute fold-change ≥ 1). **c** RNA seq data of post-mortem PFC tissues revealed 2,475 age-associated genes differentially expressed between young (<49 years of age) and old (≥49 years of age) brain samples. The Venn diagram shows that genes differentially expressed in low passage iNSCs vs. iPSC-derived low passage smNPCs include only 132 out of 2,475 genes (5.3%) differentially expressed in young vs. old PFC. **d** Scatter plot analysis of age-associated genes showing little difference between low passage iNSCs and smNPCs. **e**, **f** Expression level distribution of the 470 age-associated DEGs that are shared by all 3 comparisons (young vs. old PFC as well as low passage iNSCs and smNPCs vs. PBCs) shows no difference across low and high passage smNPCs and iNSCs. In contrast, gene expression level distribution across the young and old PFC samples shows a clear separation (Supplementary Fig. 12e, f) between the two groups. Data are shown for genes with higher expression levels in young (**e**) and higher expression levels in old (**f**) PFC samples. Box plots display the median (center line), the 25th and 75th percentile (upper and lower box, respectively) and 1.5-times the interquartile range from the box (whiskers). Data points outside this region are not shown

The profound loss of age-associated epigenetic signatures in adult blood-derived iNSCs led us to further investigate whether iNSCs also lack age-associated transcriptional signatures. RNA sequencing (RNA seq) results showed that, in line with the global DNAm profiling, PB-iNSCs exhibit global transcriptional profiles more similar to those of isogenic smNPCs than to those of their parental PBCs (Fig. 6a, b). Interestingly, iNSCs at low and high passages exhibit subtle differences in their expression profiles, which suggest ongoing transcriptional changes during long-term expansion of iNSCs (Fig. 6a, b). To assess to what extent age-associated transcriptional signatures are preserved in our iNSCs, we compared our results to RNA seq data generated from young and old post-mortem human pre-frontal cortex (PFC) tissues (0–89 years)^11^. A total of 2,475 genes were found to be differentially expressed between young (<49 years of age) and old (≥49 years of age) PFC samples and thus regarded as a proxy for age-associated genes. Strikingly, only 132 of these genes (i.e., 5.3% of all age-associated genes) were differentially expressed between iNSCs and isogenic iPSC-derived smNPCs at low passages (Fig. 6c, d). In contrast, 656 and 598 age-associated genes (26.5 and 24.2% of all age-associated genes) were differentially expressed in low passage iNSCs and smNPCs when compared to the original PBCs, respectively (Supplementary Fig. 12a). Notably, most of these age-associated transcriptional alterations (470 genes) were shared by iNSCs and smNPCs (Supplementary Fig. 12a). Within these 470 genes, there were 293 and 177 genes that were up- and down-regulated in young vs. old PFC tissue, respectively (Supplementary Fig. 12b). 321 of these 470 genes (68.3%) showed the same direction of expression change (up- or down-regulation) observed in low passage iNSCs and smNPCs vs. their parental PBCs. Specifically, 224 and 97 genes were concordantly up- and down-regulated, respectively, in young vs. old PFC and in low passage iNSCs and smNPCs vs. their parental PBCs (Supplementary Fig. 12c). In addition, we found that only 127 of the 2,475 age-associated differentially expressed genes (DEGs, 55 of the 470 genes) overlapped with genes differentially expressed in low vs. high passage iNSCs (127 out of 1,051 genes; 12.1%), suggesting that only a minority of the changes in age-associated gene expression coincides with transcriptional alterations during extensive in vitro cultivation (Supplementary Fig. 12d). Gene ontology (GO) analysis based on the 224 genes that were concordantly up-regulated in young PFC tissue as well as in low passage iNSCs and smNPCs revealed GO terms associated with categories that are likely affected during CNS aging, including pathways affecting organism behaviors (GO:0044708, GO:0007610, and GO:0030534), learning and memory (GO:0007611, GO:0007612, and GO:0007613), as well as cognition (GO:0050890). We also found that the 470 age-associated DEGs that were shared by all 3 comparisons (young vs. old PFC as well as low passage iNSCs and smNPCs vs. PBCs) displayed very similar expression levels in iNSCs and smNPCs at both low and high passages (Fig. 6e, f and Supplementary Fig. 12e, f). These data strongly indicate a substantial loss of age-associated transcriptional signatures in PB-iNSC cultures. As a result, PB-iNSCs display transcriptional aging signatures comparable to those of PSC-derived NPCs.

In addition to assessing global age-associated methylation and transcriptional signatures, we also analyzed a set of distinct cellular aging hallmarks. Previous studies had revealed that physiologically aged cells exhibit decreased expression of nuclear lamina-associated polypeptide 2α (LAP2α), impaired autophagic flux, and increased levels of cellular senescence and apoptosis^6,38–40^. We assessed these aging hallmarks in our isogenic iNSC and smNPC cultures by various experimental approaches (Supplementary Fig. 13). Immunostaining of LAP2α protein and qPCR analyses of *LAP2α* and *LMNB1* revealed no differences between the two cell populations with respect to expression of these markers. Next, we studied autophagic flux by analyzing the levels of the autophagy substrate p62 and the conversion of autophagy marker LC3 from its cytosolic form (LC3-I) to its activated form (LC3-II) in the absence and presence of the lysosomal inhibitor Bafilomycin A (BAF). While BAF treatment increased the ratio of LC3-II/LC3-I as expected, no significant differences were detected between iNSC and smNPC cultures. In addition, mRNA expression levels of the autophagy markers *ATG5* and *ATG7* showed no significant differences between iNSC and smNPC cultures. Finally, mRNA expression of the senescence- and apoptosis-associated genes *p16INK4a*, *p14ARF*, and *MMP13* was analyzed by qPCR. Again, no significant differences between iNSCs and smNPCs were detected (Supplementary Fig. 13). Taken together, these results demonstrate that adult blood-derived iNSCs are largely devoid of age-associated epigenetic, transcriptional, and cell function-related alterations.

### iNSCs are suitable for disease modeling and transplantation

In vitro disease modeling and transplantation-based cell therapies have emerged as major applications of patient-specific iPSCs^5,6^. Since iNSC generation from peripheral blood represents an even faster and very efficient route for the generation of patient-specific cells, we wondered whether our iNSCs would, in principle, be suited for these applications. As for disease modeling, we chose as an exemplar Machado–Joseph disease (MJD; syn. spinocerebellar ataxia type III, SCA3). MJD is a polyglutamine disease and the most frequent form of dominantly inherited ataxia worldwide^41^. The disease is caused by expansion of a CAG repeat in the *ATXN3* gene resulting in an expanded polyglutamine (polyQ) stretch in the C-terminus of the ataxin-3 protein. Upon cleavage of ataxin-3, this expanded polyQ stretch enhances the aggregation propensity of the C-terminal fragment, resulting in the formation of sodium dodecyl sulfate (SDS)-insoluble aggregates accumulating to neuronal intranuclear inclusions^41–43^. We wondered whether the pathognomonic aggregation phenotype of this disease could be modeled in neurons derived from iNSCs. To address this question, we subjected MJD-specific and control PB-iNSC cultures to spontaneous neuronal differentiation (Supplementary Fig. 14a-c). After 8 weeks, these cultures were subjected to biochemical analysis using fractionation with Triton-X-100 (TX100), SDS, and formic acid (FA) to detect SDS-insoluble aggregates^44^. Western blot analysis showed a prominent wild-type ataxin-3 band (~42 kDa) in the control cells and an additional band at ~60 kDa in the MJD cells reflecting the allele containing the expanded CAG repeat similar to the results of our previous study on MJD iPSC-derived neurons (Supplementary Fig. 14d)^44^. In addition, MJD but not control iNSC-derived cultures displayed prominent SDS-insoluble ataxin-3-positive complexes in the FA fraction (Supplementary Fig. 14d). These results recapitulate the protein aggregation phenotype observed in our previous iPSC-based MJD model, where ataxin-3-positive complexes could be detected in the FA fraction of MJD but not control cultures of differentiated iPSC-derived NSCs^44^. As such, they provide proof-of-concept that iNSCs lend themselves to modeling neurodegenerative diseases in vitro.

To study survival and differentiation of PB-iNSCs in vivo, we grafted them into the adult mouse brain. Ten to eleven weeks after transplantation, the cells had formed well-delineated grafts staining positive for the human-specific markers hN and STEM121 (Supplementary Fig. 15a). Double labeling of hN-stained cells with the cell type-specific markers MAP2 (neurons), GFAP (astrocytes), and NG2 (oligodendrocyte progenitors) revealed that in vivo, too, iNSCs undergo trilineage differentiation (Supplementary Fig. 15b-d). Immunostaining with the human-specific neurofilament antibody HO14 further showed that iNSC-derived neurons had extended long axonal projections into the ipsi- and contralateral corpus callosum (Supplementary Fig. 15e). Taken together, these in vivo data demonstrate that iNSCs grafted into the unlesioned adult rodent brain survive and undergo neuronal and glial differentiation. Although further detailed studies will be required to assess the neural subspecification and functionality of these cells in vivo, these results suggest that patient-specific iNSC could serve as an alternative resource for transplantation-based neural cell replacement.

## Discussion

Here, we present a robust approach to directly convert adult human blood cells into iNSCs in the absence of OCT4, which comes with significant advantages compared to previous studies: (i) being based on PB as the most accessible adult cell source, the approach largely facilitates donor recruitment, which can be extended to blood banks and thus to large numbers of samples, including those from patients^45^; (ii) the use of temperature-sensitive non-integrating SeV vectors provides a safeguard against integration of transgene and viral sequences into the iNSC genome, whereas previously reported retrovirus- or lentivirus-based approaches might entail disruption of endogenous gene expression and potential transgene reactivation^15,46^; (iii) our iNSCs exhibit robust self-renewal and multipotency at the single cell level, which indicates their bona fide NSC identity and potential amenability to genome editing; (iv) the generated iNSCs remain responsive to lineage patterning cues and efficiently give rise to glia and neuronal subtypes of both CNS and PNS, indicating an early developmental stage of this NSC population; (v) our approach does not depend on OCT4, which is essential to induce pluripotency in human somatic cells and has recently been implicated in inducing a transient pluripotent stage in the context of somatic-to-NPC conversion, thus challenging the concept of a direct cell fate conversion^17,19,20^.

Interestingly, global DNAm profiling revealed further unique features of our SOX2/c-MYC-induced iNSCs with regard to age-related epigenetic signatures. Three independent models employing age-specific methylation changes in different CpG sets showed that newly generated iNSCs had largely lost age-associated DNAm patterns of their parental PBCs. All three models were applied without further cell type-specific adaptation and assigned the highest DNAm age to the donor PBCs, whereas PSCs and PSC-derived NPCs were allocated as embryonic or neonatal. Age prediction was most accurate in the Horvath model, which allocated the DNAm age of the PBCs close to the chronological age of the donors and that of PSCs and their derivatives close to 0 years of age, whereas the calculated ages of iNSCs at low passages ranged between 5.5 and 39.4% of the chronological age of the donor PBCs. The accuracy of the Horvath model might be due to the fact that it was established based on multiple tissues and cell types (including CNS), whereas the Hannum and 99CpGs models were trained only with blood cells^34–36^. As the employed aging models were originally established with samples encompassing large cell numbers, we included not only single colony-derived iNSCs but also iNSC lines generated from multiple neuroepithelial colonies in order to reflect DNAm ages of the entire populations. In fact, clonally derived cell preparations may specifically capture the epigenetic age of the colony forming cell, whereas DNAm values are usually measured as average of multiple subsets^47^. Cells at low passages of both single- and poly-colony origin showed a pronounced but incomplete loss of age-related CpG methylation patterns, which further supports the notion that during SOX2/c-MYC-induced conversion of PBCs into iNSCs the cells do not transit through an intermediate iPSC-like state.

Epigenetic rejuvenation of the iNSCs is also supported by the RNA seq data, where genes differentially expressed in iNSCs and iPSC-derived smNPCs showed only a minor overlap with genes differentially expressed in young vs. old human PFC^11^. In comparison to the parental PBCs, low passage iNSCs and smNPCs shared the majority of age-associated transcriptional changes, which were also largely concordant with the expression differences observed in young vs. old human PFC tissue. The lack of complete concordance is in line with the fact that some age-associated genes expressed in the PFC tissues are not expressed or expressed at much lower/higher level in the PBCs. Accordingly, this set of genes is not expected to follow the same fold change trends defined in young vs. old PFC tissues. In addition, we observed a small overlap of DEGs between low vs. high passage iNSCs and old vs. young PFC tissue, suggesting mild alterations in the transcription of age-associated genes in iNSC cultures during long-term expansion. This is consistent with the results of our DNAm analysis, where we observed a profound loss of age-associated DNAm signatures during the PBC-to-iNSC conversion, but also a further erosion of age-associated epigenetic makeups after extensive in vitro cultivation.

The notion of an epigenetic rejuvenation across PBC-to-iNSC conversion is further supported by our finding that PB-iNSCs and their isogenic iPSC-derived counterparts show no significant differences with respect to a number of cellular aging hallmarks including LAP2α expression, autophagic flux as well as expression of senescence and apoptosis markers.

Results of previous studies indicate that continuous passaging of mouse and human iPSCs attenuates the epigenetic memories of their somatic cell origin and age^48,49^. Although the major loss of DNAm age in our iNSC paradigm occurs during the process of cell fate conversion, we also noticed that expansion and passaging of newly generated iNSCs led to a further decrease of DNAm age. The mechanisms underlying this phenomenon remain unknown, and we can currently not exclude that the transient cultivation at elevated temperature required to eliminate the SeV vectors contributes to the observed erosion of DNAm age. It is, however, noteworthy that long-term expanded iPSCs propagated in a standard 37 °C environment, too, have been reported to display an erosion of age-associated epigenetic signatures^49^. Yet, using the Horvath model, high passage iNSCs still showed DNAm ages around 5 years, whereas, in the same model, iPSCs have been shown to display DNAm ages around 0 already at low passages. These observations indicate that the major loss of DNAm age is not simply a result of cell division.

Interestingly, iNSC subclones derived from single colonies displayed discrete differences in DNAm age. This could suggest that, in contrast to iPSC generation, where epigenetic signatures of all cells are reset to the ground state^6,34,35,50^, in OCT4-free conversion each individual iNSC undergoes an independent epigenetic reprogramming process. Alternatively, this variability might be due to differences in epigenetic memory erosion during the subcloning process or to a selection for cells with a younger DNAm signature.

Stable self-renewal, the potential to differentiate into various neuronal and glial subtypes, and their efficient generation from an easily accessible source could make blood-derived iNSCs a highly attractive resource for both, disease modeling and transplantation-based regenerative approaches. For disease modeling, we resorted to MJD, a polyglutamine disorder we had previously studied in an iPSC-based system to model pathological protein aggregation in vitro. In that study we had shown that MJD but not control neurons form ataxin-3 microaggregates in culture, a phenomenon which is triggered by excitation-dependent intracellular Ca^2+^ increase, subsequent activation of calpains and cleavage of ataxin-3^44^. When we studied iNSC-derived neuronal cultures from an MJD patient already enrolled in our earlier iPSC study^44^, we could recapitulate this phenotype showing prominent accumulation of ataxin-3-positive species in the formic acid fraction.

Amenability to in vivo transplantation was assessed by grafting the cells into the unlesioned adult mouse brain, where they formed well-delineated grafts, underwent differentiation into neurons and glia, and generated long-range axonal projections. While the full potential of iNSCs for disease modeling and neural transplantation might be uncovered in the context of future studies, these proof-of-principle data indicate that iNSCs lend themselves to both applications.

Taken together, our study indicates that adult blood-derived iNSCs might develop into a particularly attractive resource for disease modeling and regenerative medicine.

## Methods

### Ethics Statement

Primary PB samples were collected from healthy donors and MJD patients at different ages. The study complies with all relevant ethical regulations and was approved by the ethics committee of the University of Bonn Medical Center (approval number: 275/08). All subjects gave written informed consent. CB-CD34^+^ cells were provided by the Institute for Transplantation Diagnostics and Cell Therapeutics, Heinrich-Heine University of Düsseldorf with written informed consent. Human fetal brain RNA used in this study was kindly provided by the MRC/Welcome Trust funded Human Developmental Biology Resource at Newcastle University with appropriate maternal written consent and approval from the Newcastle and North Tyneside NHS Health Authority Joint Ethics Committee.

### Generation of iNSCs from PBCs and CBCs

For PBC preparation, PB was separated in CPT tubes (BD Vacutainer CPT) following the manufacturer’s protocol to isolate peripheral blood mononuclear cells (PBMCs). In brief, PBMCs were seeded at 5–7 × 10^6^ cells/ml and cultured in erythroblast expansion medium: StemSpan SFEM (Stem Cell Technologies) supplemented with 100 ng/ml SCF (R&D), 2 U/ml EPO (R&D), 40 ng/ml IGF-1 (R&D), 10 ng/ml IL-3 (R&D), 1% chemically defined lipid concentrate (Life Technologies), and 1 µM dexamethasone (Sigma). After 7 days, erythroblasts were purified by a Percoll density gradient (Sigma)^23^. Purified erythroblasts were expanded for another 2 days in expansion medium without IL-3 at a density of 1–1.5 × 10^6^ cells/ml with daily medium replacement. CB-CD34^+^ cells were cultured for 1 day before infection in CBC medium: DMEM/F12 (Life Technologies), 30% fetal calf serum (FCS, Life Technologies), 1% penicillin–streptomycin (Life Technologies), 2 mM L-glutamine (Life Technologies), 50 ng/ml SCF (R&D), 10 ng/ml TPO (PeproTech), 20 ng/ml Flt-3 ligand (PeproTech), and 50 ng/ml IL-6 (PeproTech).

Erythroblasts and CB-CD34^+^ cells were infected with SeV expressing SOX2 (MOI ≈ 3.9–5) and c-MYC (MOI ≈ 1–5) (CytoTune^TM^-iPS kit, ThermoFisher) at day 0. In detail, 3 × 10^5^ erythroblasts or 1 × 10^5^ CB-CD34^+^ cells were spin-infected in a 2 ml Eppendorf tube for 30 min at 1,200*g* and 32 °C in IL-3-free erythroblast expansion medium or CBC medium containing SeV-SOX2 and SeV-c-MYC. Cells were then re-suspended without changing the medium and incubated for another 24 h at 37 °C, 21% O_2_, 5% CO_2_. At day 1, infected cells were replated into dishes coated with matrigel (Corning) in iNSC conversion medium: N2B27 (advanced DMEM/F12:Neurobasal (both from Life Technologies) (1:1), 1× N2 (GE Healthcare), 1× B27 (without vitamin-A, Life Technologies), 2 mM L-glutamine) plus 3 μM CHIR99021 (Miltenyi), 0.5 μM A83-01 (Tocris) (alternatively 2 μM SB431542, Tocris), 0.5–1 μM purmorphamine (Tocris), 10 ng/ml hLIF (Novoprotein), 64 μg/ml L-ascorbic-acid-2-phosphate (LAAP, Sigma), and 5 μM tranylcypromine (Tocris, only present in the first 10 days). For initial experiments, infected cells were plated on MEFs. For iNSC generation, cultures were maintained under hypoxic conditions (37 °C, 5% O_2_, 5% CO_2_). Single neuroepithelial colonies were manually picked between day 11 and day 21 and further expanded as single colony-derived iNSCs. Alternatively, neuroepithelial colonies were re-suspended using pre-warmed accutase (Life Technologies) and cultured as poly-colony-derived iNSCs. The iNSC conversion efficiency was calculated as the number of neuroepithelium-like colonies counted at day 14 divided by the number of SeV-SOX2/c-MYC infected PBCs replated at day 1 in one infection experiment. Single- and poly-colony-derived iNSCs were further propagated in matrigel-coated dishes in iNSC expansion medium: N2B27 supplemented with 3 μM CHIR99021, 0.5 μM A83-01 (or 2 μM SB431542), 0.5 μM purmorphamine, 10 ng/ml hLIF, and 64 μg/ml LAAP. Cultures were switched back to normoxic conditions (37 °C, 21% O_2_, 6–9% CO_2_) once iNSC colonies attached. After expansion for 7–10 passages, iNSCs were cultured at 39 °C for about 3–4 weeks until the complete elimination of SeV vectors was confirmed by RT-PCR.

For routine expansion, iNSCs were typically split using a 1:3 to 1:5 ratio every 4–5 days. For splitting, cultures were suspended into single cells with pre-warmed accutase for about 10 min at 37 °C, diluted with DMEM/F12, and centrifuged at 1,000 rpm for 5 min. The cell pellets were re-suspended in fresh iNSC expansion medium and replated in matrigel-coated dishes.

### Clonal analysis of iNSCs

iNSCs were first stained with 1 µg/ml Hoechst 33342 (Sigma), then single cells were deposited into matrigel-coated 96-wells using FACS and subsequently cultured in iNSC expansion medium. On day 0, the initial single-cell status was confirmed. Phase-contrast image analysis was performed every 2 days until day 12 using the IN Cell Analyzer system (GE Healthcare) to monitor cell growth in each well. Expandable single iNSC subclones were passaged after 2 weeks and expanded for further experiments.

### Differentiation of iNSCs

For spontaneous differentiation, iNSCs were seeded in matrigel-coated dishes and cultured in N2B27 medium (DMEM/F12:Neurobasal (1:1), 0.5× N2, 0.5× B27, 2 mM L-glutamine) supplemented with 64 μg/ml LAAP, 10 ng/ml BDNF (CellGS), 10 ng/ml GDNF (CellGS), and 0.5 mM dibutyryl cAMP (dbcAMP, Enzo Life Sciences). For immunocytochemistry and qPCR, iNSCs were spontaneously differentiated for 3–6 weeks with 5 µM DAPT (Tocris), and the medium was changed every 2–3 days. For DNAm analysis, iNSCs were differentiated for 8 weeks with 1% penicillin/streptomycin (Life Technologies). For electrophysiological measurements, iNSCs were differentiated on mouse astrocytes for 8–12 weeks with 1% penicillin/streptomycin^10^.

For induction of motoneurons, iNSCs were cultured in N2B27 medium plus 64 µg/ml LAAP, 0.5 µM purmorphamine, 1 µM CHIR99021, 2 µM SB431542, 2 µM DMH1 (Tocris), 0.1 µM retinoic acid (Sigma), 2 µg/ml laminin, and 2 µg/ml fibronectin (Life Technologies) for 8 days. At day 8, cells were split 1:2 to 1:3 with accutase and 10 µM Y-27632 (Tocris), and replated in matrigel-coated dishes. From day 9 to day 13, patterned iNSCs were differentiated in N2B27 medium with 0.5 µM retinoic acid, 0.1 µM purmorphamine, 64 μg/ml LAAP, 2-4 µg/ml laminin, and 2–4 µg/ml fibronectin. In the following maturation phase, 20 ng/ml BDNF, 20 ng/ml GDNF, 20 ng/ml IGF-1 (R&D), 0.5 mM dbcAMP, and 0.1 µM Compound E (Tocris) were added to the medium. The medium was changed every 2 days, and cells were collected at day 8 and day 25 for immunocytochemistry and qPCR.

For generation of midbrain dopamine-like neurons, iNSCs were cultured in modified N2B27 medium (DMEM/F12:Neurobasal (1:1), 1× N2, 1× B27 (both decreased to 0.5× after day 4) and 2 mM L-glutamine) plus 100 ng/ml SHH-C24 II (Novoprotein), 1 μM purmorphamine, 1 μM SAG, and 0.7 μM CHIR99021 for 8 days. 100 ng/ml FGF8b (PeproTech) was added to the media from day 0 to day 10. At day 10, cells were split 1:2 to 1:3 with accutase and 10 µM Y-27632, and replated in matrigel-coated dishes. From day 10 to day 14, cells were differentiated in N2B27 medium with 10 ng/ml BDNF, 10 ng/ml GDNF, 64 μg/ml LAAP, 2-4 µg/ml laminin, and 2–4 µg/ml fibronectin. From day 14, 0.5 mM dbcAMP and 5 µM DAPT were added to the medium. The medium was changed every 2 days, and cells were collected at day 10 and day 25 for immunocytochemistry and qPCR.

For oligodendrocyte differentiation, a 3-stage-protocol was applied^26^. First, 90,000 cells/cm^2^ were plated in matrigel-coated dishes in modified N2 medium (DMEM/F12, 1× N2, 1.6 g/l D-glucose, 0.2 mg/ml apo-transferrin (Millipore), 0.02 mg/ml insulin (Sigma)) plus 10 ng/ml EGF, 10 ng/ml PDGF-AA (R&D), and 10 mM forskolin (Sigma) (stage I). After 2 weeks, cells were switched to modified N2 medium supplemented with 10 ng/ml PDGF-AA, 30 ng/ml T3 (Sigma), 200 ng/ml noggin (R&D), and 200 mM ascorbic acid (Sigma) for 1 week (stage II). The first two steps were performed to support the development and proliferation of oligodendroglial progenitors. Terminal differentiation into oligodendrocytes (stage III) was induced by growth factor withdrawal. 30 ng/ml T3, 200 mM ascorbic acid, and 1 μg/ml laminin were added to the modified N2 medium for at least 4 weeks before immunocytochemistry. At each differentiation stage, half of the medium was changed every 2–3 days.

### Generation of PB-iPSCs from PBCs

3 × 10^5^ erythroblasts were infected with Sendai virus (CytoTune™-iPS 2.0 Sendai Reprogramming Kit, ThermoFisher) and incubated for 24 h. Transduced cells were harvested, re-suspended in fresh IL-3-free erythroblast expansion medium, and cultured for another 2 days. Cells were then collected and seeded in dishes coated with Geltrex (ThermoFisher) and cultured in hypoxic atmosphere (5% O_2_ and 5% CO_2_). The day after plating, cultures were transferred to non-supplemented SFEM. Three days later, the medium was changed to a 1:1 mixture of non-supplemented SFEM and E7 medium for 2 days, and then switched to 100% E7 with daily medium changes. PB-iPSC colonies were isolated between days 17 and 23 post-infection and expanded in mTeSR1 (STEMCELL Technologies) on Geltrex.

### Differentiation of PB-iPSCs into smNPCs

Generation of smNPCs from PB-iPSCs is based on a published protocol^25^. PB-iPSCs were first dissociated with accutase and seeded in AggreWells (STEMCELL Technologies) for embryoid body (EB) formation. Cells were cultured in N2B27 medium with 10 µM Y-27632, 0.5 µM purmorphamine, 3 µM CHIR99021, 10 µM SB431542, and 1 µM dorsomorphin (Tocris). After 3 days, EBs were transferred to 6 cm dishes in N2B27 medium with 0.5 µM purmorphamine, 3 µM CHIR99021, 10 µM SB431542, and 1 µM dorsomorphin. At day 4 or 5, the medium was changed to N2B27 supplemented with 64 μg/ml LAAP, 0.5 µM purmorphamine, 3 µM CHIR99021, and the same medium condition was applied thereafter. At day 6, EBs were triturated into small pieces, plated in matrigel-coated dishes, and smNPCs were passaged at a ratio of 1:5 to 1:10.

### SNP analysis

Genomic DNA was prepared using the DNeasy Blood & Tissue Kit (Qiagen). Whole-genome single nucleotide polymorphism (SNP) genotyping was performed at the Institute of Human Genetics at the University of Bonn. Genomic DNA at a concentration of about 50 ng/μl was used for whole-genome amplification. Afterwards, the amplified DNA was fragmented and hybridized to sequence-specific oligomers bound to beads on an Illumina GSAMD-24v1-0 chip (Illumina). Data were analyzed using Illumina GenomeStudio V2011.1 (Illumina).

### Telomere length analysis

Measurement of median telomere length of PBC, iNSC, iPSC, and smNPC cultures was performed by Life Length, Madrid, Spain, using their proprietary Telomere Analysis Technology (TAT^®^), a high-throughput technique based on a quantitative fluorescence in situ hybridization (Q-FISH) modified for cells in interphase^51^. Briefly, cells were seeded in clear bottom black-walled 384-well plates at a density of 15,000 cells per well with 5 replicates for each sample. High-throughput Q-FISH was then performed, and the fluorescence intensities were translated to base pairs through a standard regression curve, which is generated using control cell lines of known telomere length. The average of median telomere length (base pairs) for each cell population was calculated in GraphPad Prism 6. Statistical analyses were performed by one-way ANOVA followed by Tukey's multiple comparison test.

### Immunofluorescence analysis

Cells were fixed in 4% neutral-buffered para-formaldehyde (PFA, Sigma) for 10 min at room temperature. For detection of γ-aminobutyric acid (GABA), 0.1% glutaraldehyde (Sigma) was included in the fixative. Cells were blocked and permeabilized with 10% FCS and 0.5% Triton X-100 (Sigma) in PBS for 1–2 h. Samples were incubated with primary antibodies at 4 °C overnight, washed three times, incubated with secondary antibodies at room temperature for 1–2 h, counterstained with DAPI, and mounted with mounting solution. Images were taken using ZEISS Imager Z1 or Observer Z1 microscope. Non-destructive image acquisition and handling was performed using the image acquisition software ZEN (ZEISS). All antibodies used in this study are listed in Supplementary Table 3.

### RNA extraction, RT-PCR, and quantitative RT-PCR (qPCR)

Total RNA was isolated from cell cultures using Trizol (Life Technologies) or RNeasy Mini Kit (Qiagen), and quantified by a NanoDrop spectrophotometer (Thermo Scientific). Isolated RNA was reverse-transcribed using iScript cDNA Synthesis Kit (BIO-RAD) or qScript cDNA Synthesis Kit (Quanta Biosciences) according to the manufacturer’s instructions. qPCR analyses were performed with miScript SYBR Green PCR Kit (Qiagen) on an Eppendorf Mastercycler. Cycling conditions were 40 cycles of 95 °C for 15 s, 60 °C for 20 s, and 72 °C for 30 s. All data were normalized to *18s* rRNA levels. PCR products were assessed by dissociation curve and gel electrophoresis. Normalized data were analyzed using the ΔΔCt method, calculated in Microsoft Excel and further processed in GraphPad Prism 6. Semi-quantitative RT-PCR analyses were performed using Taq Polymerase (Life Technologies) according to the manufacturer’s instructions. Cycling conditions were 30–35 cycles of 94 °C for 30 s, 60 °C for 30 s, 72 °C for 30–60 s; for *18s* primers parameters were 25 cycles of 94 °C for 30 s, 60 °C for 30 s, 72 °C for 60 s. Primers used in this study are listed in Supplementary Table 4.

### Electrophysiology

Whole-cell current-clamp and voltage-clamp recordings were carried out with an Axopatch-200B amplifier (Molecular Devices) interfaced by an A/D-converter (Digidata 1440, Molecular Devices) to a PC running PClamp software (Version 10, Molecular Devices). Pipette electrodes (GB150F-8P, Science Products) were fabricated using a vertical puller (Narishige PC-10) and filled with a solution containing (in mM): 120 potassium gluconate (C_6_H_11_O_7_K), 20 KCl, 10 NaCl, 10 EGTA, 1 CaCl_2_, 4 Mg-ATP, 0.4 Na-GTP, 10 HEPES (pH 7.2; 280–290 mOsm). In addition, the pipette solution also contained 0.1% biocytin, and neurons were filled by passive diffusion of biocytin from the patch pipette during electrophysiological recording. The signals were low-pass filtered at 10 kHz and sampled at 50 kHz. All recordings were performed at room temperature (22–25 °C) in a bath solution containing (in mM): 140 NaCl, 5 KCl, 2 CaCl_2_, 1 MgCl_2_, 10 HEPES and 25 D-glucose (pH 7.2; 310–320 mOsm). The equilibrium potential of chloride ions in these recording conditions was around −40 mV. sEPSCs and sIPSCs were recorded at a holding potential of −70 and 0 mV, respectively.

### Microarray-based gene expression analysis

Global gene expression profiles were analyzed by HumanHT-12v4 Expression BeadChip kit (Illumina). The raw data were pre-processed and normalized using illuminaio^52^ and limma^53^ packages in R. All further calculations were performed on log2 transformed data. PCA and pairwise scatterplots were performed using R. The expression profiles of selected genes were subjected to hierarchical analysis and visualized in heat map format.

### Global DNA methylation analysis

DNAm profiles were analyzed using the Infinium HumanMethylation450 BeadChip (Illumina), which addresses 485,512 CpG dinucleotides at a single-nucleotide resolution. The raw DNAm data were analyzed using minfi package in R^54^. The publicly available DNAm data were retrieved from GSE38216 and GSE40790 in NCBI GEO^32,33^. To estimate the age of the samples based on DNAm profiles, three epigenetic age predictor models (Horvath, Hannum, and 99CpGs models) were utilized in the analysis^34–36^.

### 3′ mRNA sequencing (RNA seq) analysis

RNA samples were extracted using the RNeasy Mini Kit. RNA Seq libraries were prepared using the QuantSeq 3′ mRNA seq Library Prep Kit (Lexogen) according to the manufacturer’s instructions. Libraries were sequenced using the Illumina HiSeq 2500 platform. Transcript and gene level quantification was performed using Salmon^55^ in combination with the human cDNA file for genome GRCh37 version 89 obtained from ENSEMBL. Gene level read counts were then further analyzed in R using tximport and DESeq2. Prior to further analysis, mitochondrial genes, as well as genes with less than a total read count of 4 across all samples (40th percentile) and more than 17,488 reads (95th percentile) were removed. All data were normalized based on standard size factor estimates, and differential expression analysis was performed using a Negative Binomial generalized linear and the corresponding Wald test. Resulting *P*-values were corrected for multiple testing using Benjamin–Hochberg correction. Only genes with an adjusted *P*-value below 0.05 and minimum log2 fold change greater than 1 were considered to be differentially expressed between conditions.

For the analysis of previously published RNA seq data from post-mortem dorso-frontal cortex samples, we downloaded raw fastq files from the ERP008608 ArrayExpress submission. Subsequently, we performed identical pre-processing steps as described above. In order to identify a gene set enriched for age-related genes, we simply divided the 14 samples into two groups, one comprising all samples obtained from ages <49 years and one with all remaining samples. Subsequently, we again performed differential expression analysis similar to the strategy described above, classifying all genes below an adjusted *P*-value of 0.01 as differentially expressed. We regarded these genes as a proxy for genes that change in an age-dependent manner in subsequent analyses. All heatmaps, clustering, etc. were generated using the R-statistical analysis framework.

### Assessment of autophagic activity

Isogenic iNSCs and smNPCs were treated with 40 nM Bafilomycin A1 (BAF, Enzo Life Sciences) at 37 °C for 4 h. Treated cells and untreated controls were collected in ice-cold PBS and centrifuged at 500*g* for 5 min at 4 °C. Cell pellets were directly processed for cell lysis or frozen in liquid N_2_ and stored at −80 °C. Pellets were lysed in 25 mM Tris–HCl (pH 7.6), 150 mM NaCl, 1% NP-40, 1% sodium deoxycholate, and 0.1% SDS supplemented with protease and phosphatase inhibitor cocktail (ThermoFisher). 20 µg protein lysate per sample were separated on self-made 15% SDS-acrylamide resolving gels (+4% acrylamide stacking gels) and transferred on Immun-Blot PVDF membranes (0.2 µm; BIO-RAD) by wet blotting. Membranes were blocked in 10% milk powder (Roth) in 1× TBS-T. Primary and secondary antibodies were diluted in 5% milk powder solution. Luminata Classico (Millipore) and Luminata Crescendo (Millipore) were used to detect HRP-signal using the ChemiDoc XRS+ with Image Lab software (BIO-RAD). Quantitative image analysis was performed with ImageJ (1.47v) software. For statistical analysis of the qPCR and Western blot results shown in Supplementary Fig. 13, data were tested for normal distribution with Kolmogorov–Smirnov test. Since the assumption of normal distribution was not met, non-parametrical testing was conducted. Wilcoxon signed rank test and Kruskal–Wallis non-parametric ANOVA were performed to compare two or more groups, respectively. The significance level was set to 0.05. All statistical analyses were run with R 3.3.2 software (GUI 1.68, Mavericks build).

### Statistical analysis

Data are presented as mean + s.d. Methods for statistical analyses are described in the respective sections above.

## Electronic supplementary material

Supplementary Information

## Data Availability

Data are available from the authors upon request. The accession numbers of the public DNA methylation data are GSE38216 and GSE40790 from NCBI GEO. The accession number of the public RNA sequencing data is ERP008608 from ArrayExpress. The accession numbers for the data reported in this paper are GEO GSE90129 (microarray data) and GEO GSE117720 (RNA seq data).
