# Supplementary material for: A stably self-renewing adult blood-derived induced neural stem cell exhibiting patternability and epigenetic rejuvenation
Source: Nat Commun. 2018 Oct 2;9:4047. doi: 10.1038/s41467-018-06398-5 (PMC6168501; doi:10.1038/s41467-018-06398-5)
Supplement: Supplementary file 1 — Supplementary Information [file 41467_2018_6398_MOESM1_ESM.pdf]

Supplementary Information

A stably self-renewing adult blood-derived induced neural stem cell exhibiting patternability and epigenetic rejuvenation

Sheng et al.

Supplementary Figure 1

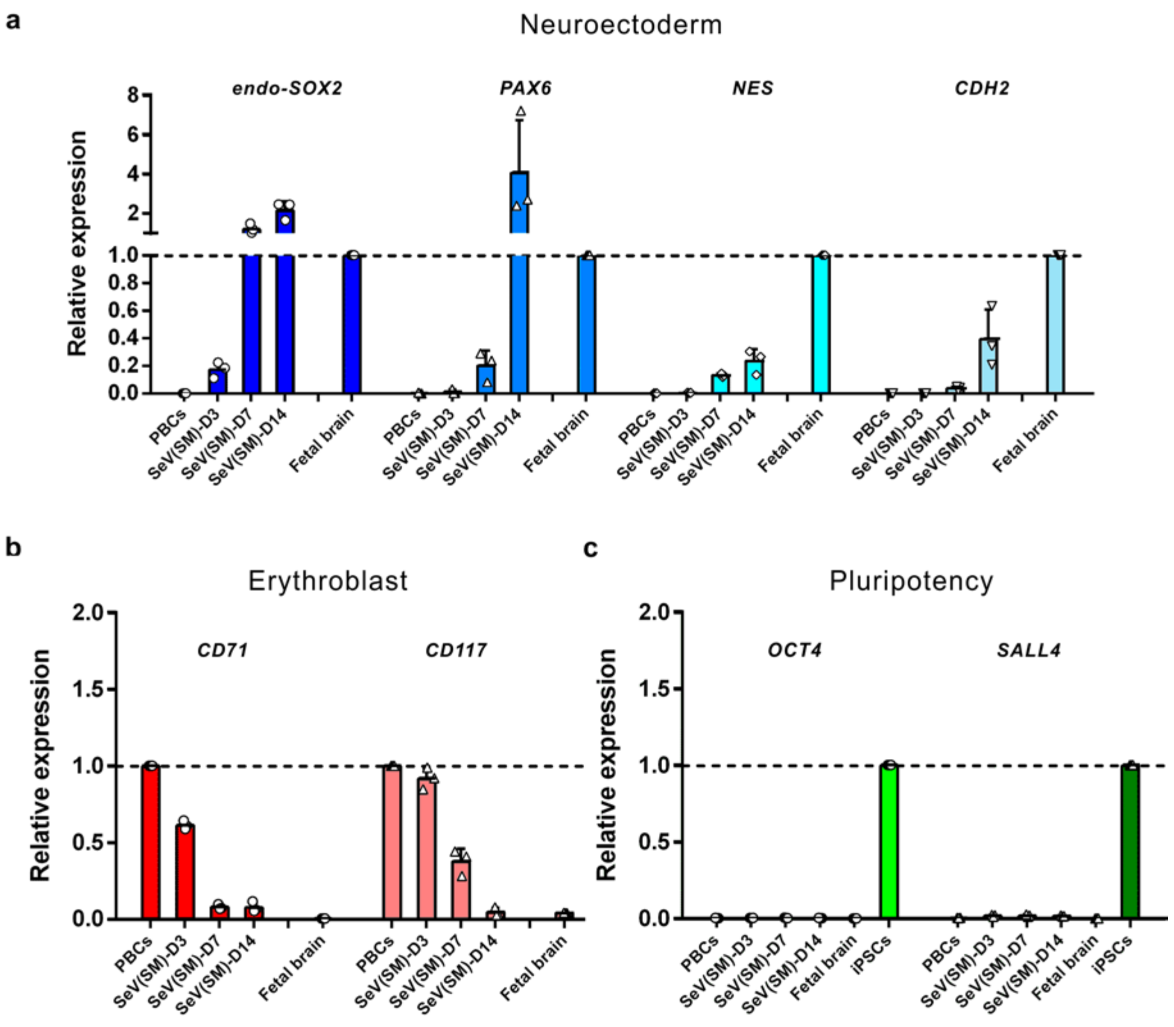

Supplementary Figure 1. Expression of neuroectoderm-, blood-, and pluripotency-associated genes during iNSC conversion. (**a**) Expression of the neuroectodermal markers *SOX2* (endogenous), *PAX6*, nestin (*NES*), and *CDH2* is strongly and continuously upregulated within the first 2 weeks of iNSC conversion. (**b**) Expression of *CD71* and *CD117*, markers of the erythroblast lineage, decreases rapidly during the early phase of iNSC generation. (**c**) The key pluripotency gene *OCT4* and the reprogramming-associated gene *SALL4* are not detectable during iNSC conversion. Data are represented as mean + s.d.; n=3. SeV(SM)-D3, D7, D14: 3, 7, 14 days after infection with SeV-SOX2 and SeV-c-MYC.

Supplementary Figure 2

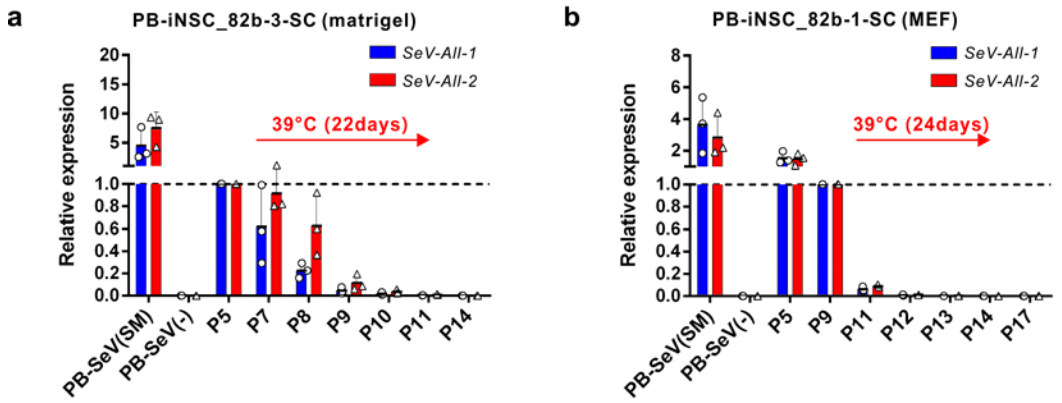

Supplementary Figure 2. iNSCs are SeV-free after 39°C incubation. (**a, b**) qPCR analyses with two pairs of SeV backbone-specific primers (*SeV-all* primers) reveal the fast decline of SeV expression during cultivation at 39°C and the persistent absence of SeV genomes upon further expansion in iNSC cultures generated on matrigel or MEF. Data are represented as mean + s.d.; n = 3.

Supplementary Figure 3

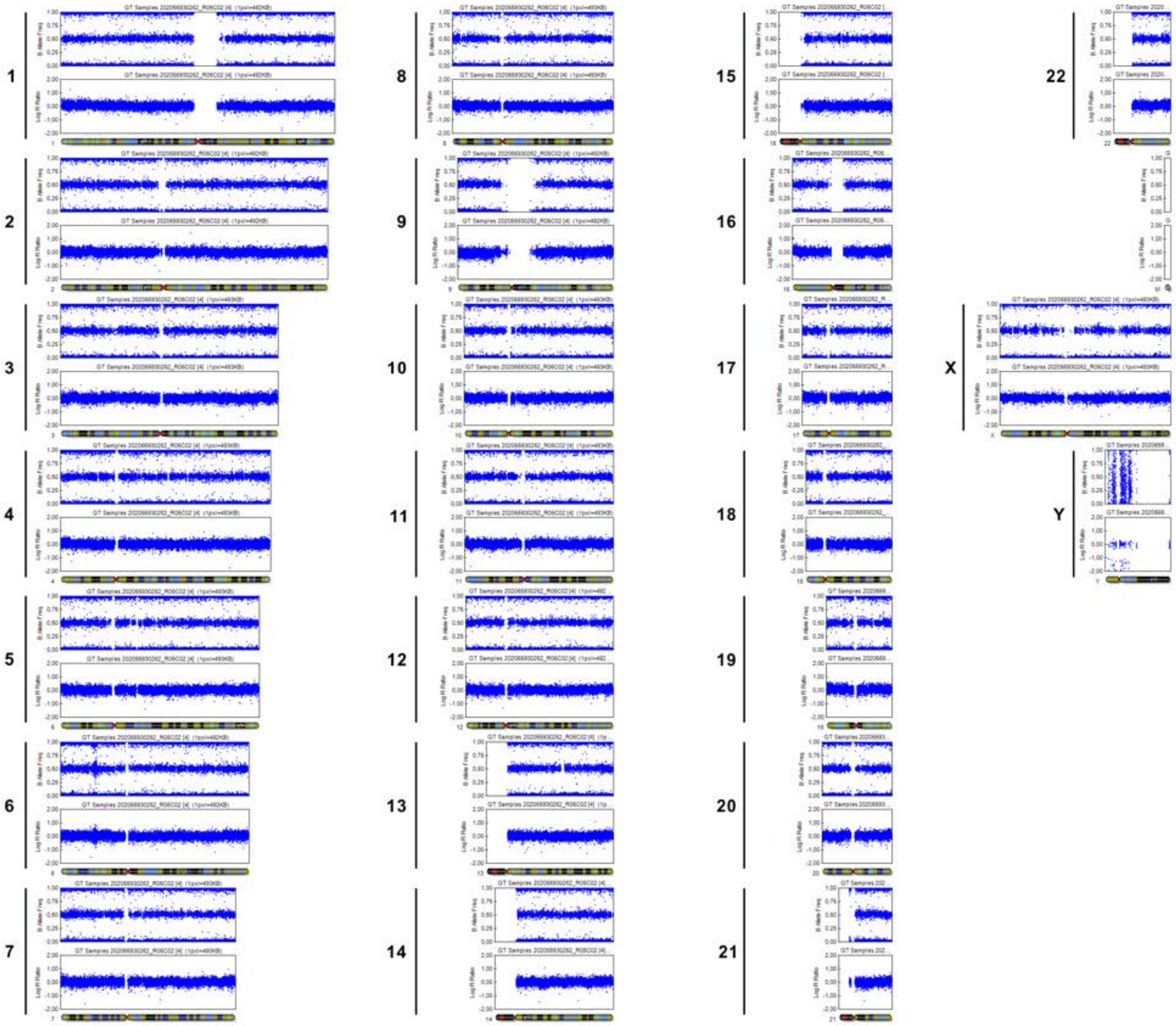

Supplementary Figure 3. Transgene-free iNSCs display normal karyotype. Genomic DNA of one single-colony-derived PB-iNSC line (82b-3-SC passage 21) was isolated at indicated passage number and subjected to single nucleotide polymorphism analysis. For each chromosome the B allele frequency (upper row) and the log R ratio (lower row) are shown.

Supplementary Figure 4

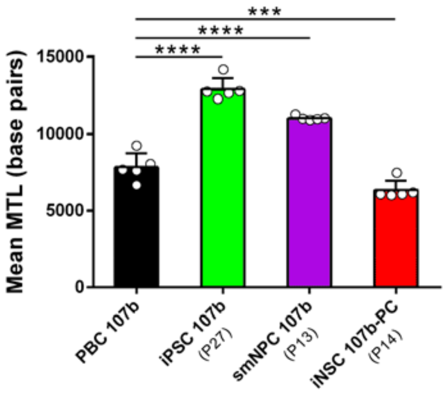

Supplementary Figure 4. iNSCs lack telomere elongation. The median telomere length (MTL) of each cell population was measured in 5 replicates by high throughput Q-FISH. The bar graph shows the mean MTL of isogenic PBCs, iNSCs, iPSCs, and smNPCs at the indicated passage numbers. Data are represented as mean + s.d.; n = 5. *** $p<0.001$, **** $p<0.0001$: One-way ANOVA followed by Tukey's multiple comparison test.

Supplementary Figure 5

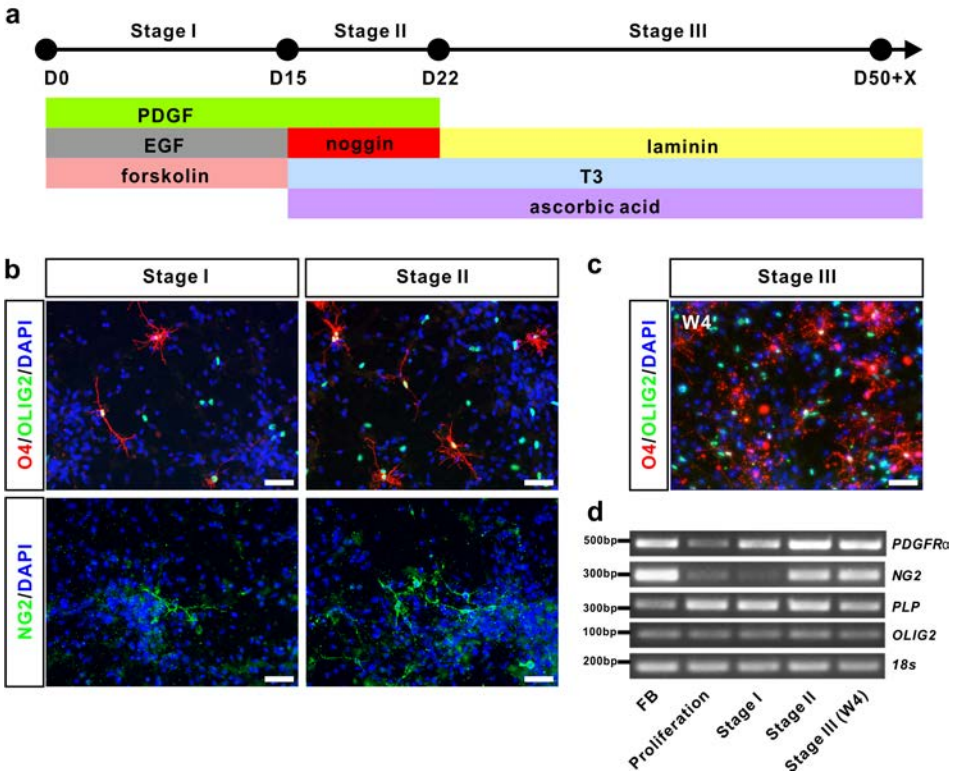

Supplementary Figure 5. Oligodendrocyte differentiation of PB-iNSCs. (**a**) Schematic representation of the 3-stage-protocol used for the differentiation of PB-iNSCs into oligodendrocytes. (**b**) Expression of the oligodendroglial markers NG2, O4, and OLIG2 at stages I and II of differentiation. (**c**) After 4 weeks of terminal differentiation in stage III (D50 of the protocol), cells exhibited complex oligodendroglial morphologies with co-expression of O4 and OLIG2. Around 12 to 16% of the cells were found to express O4 in cultures differentiated from two iNSC lines. (**d**) RT-PCR analysis of oligodendroglial markers *PDGFRα*, *NG2*, *PLP*, and *OLIG2* at different stages of oligodendrocyte differentiation. Stage III (w4): 4 weeks differentiation at stage III; FB: fetal brain. Scale bars: 50 µm.

Supplementary Figure 6

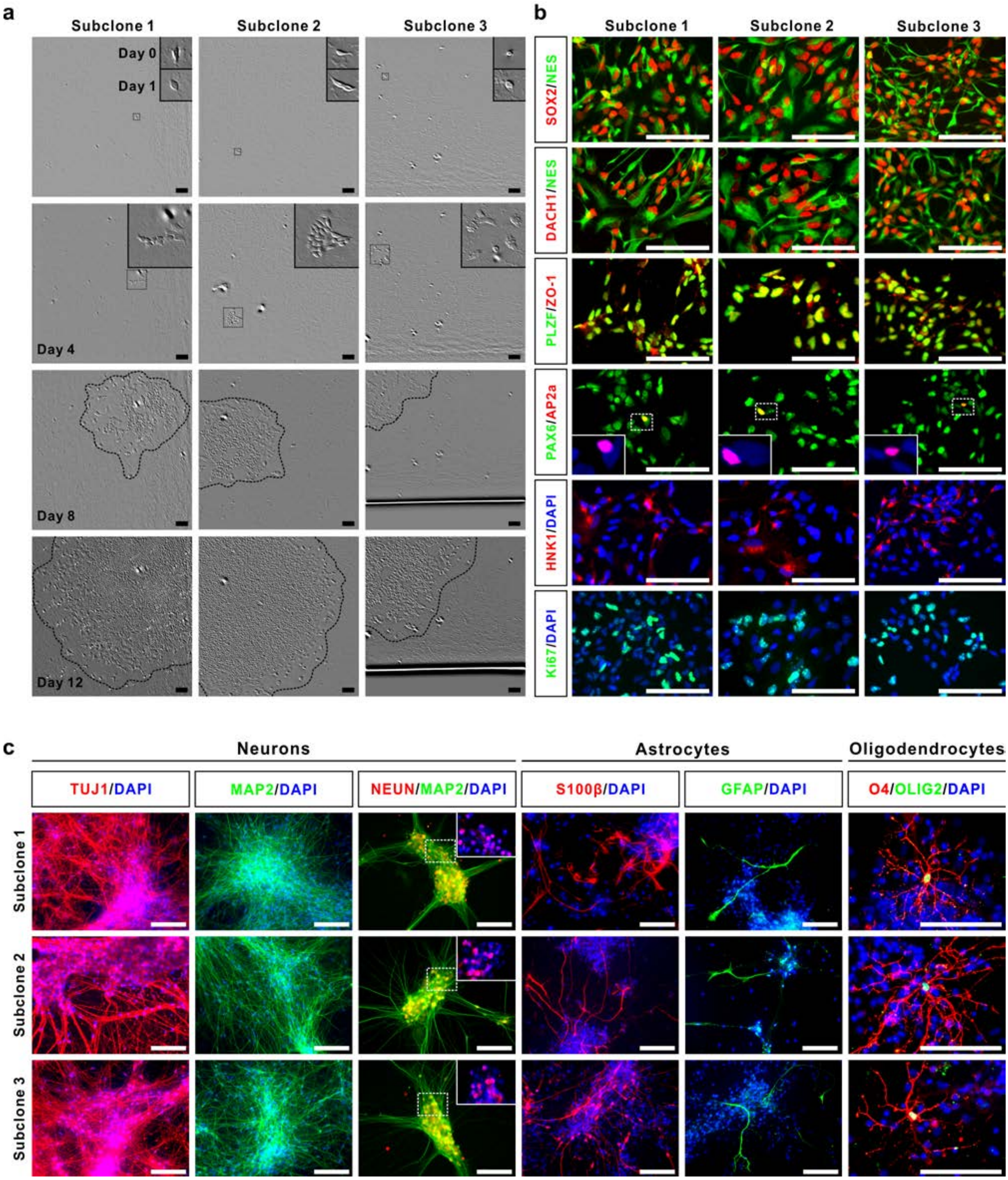

Supplementary Figure 6. iNSCs are stably self-renewing and tripotent at the single cell level. (**a**) After FACSorting of a single-colony-derived iNSC population (82b-3-SC), 13 wells contained a single iNSC as confirmed by microscopy; 8 of these single cells re-established clonal lines (clonal efficiency: 61.5%). The initial expansion of iNSC subclones is depicted in 3 randomly chosen wells. (**b**) iNSC_82b-3-SC subclones

express markers characteristic for early neuroectoderm and neural rosettes (SOX2, NES, DACH1, PLZF, ZO1, and PAX6), neural crest (AP2α, HNK1), and proliferation (Ki67). (**c**) iNSC subclones are tripotent and differentiate into neurons (TUJ1, MAP2, NEUN), astrocytes (S100β, GFAP), and oligodendrocytes (O4, OLIG2). Scale bars: 100 μm.

Supplementary Figure 7

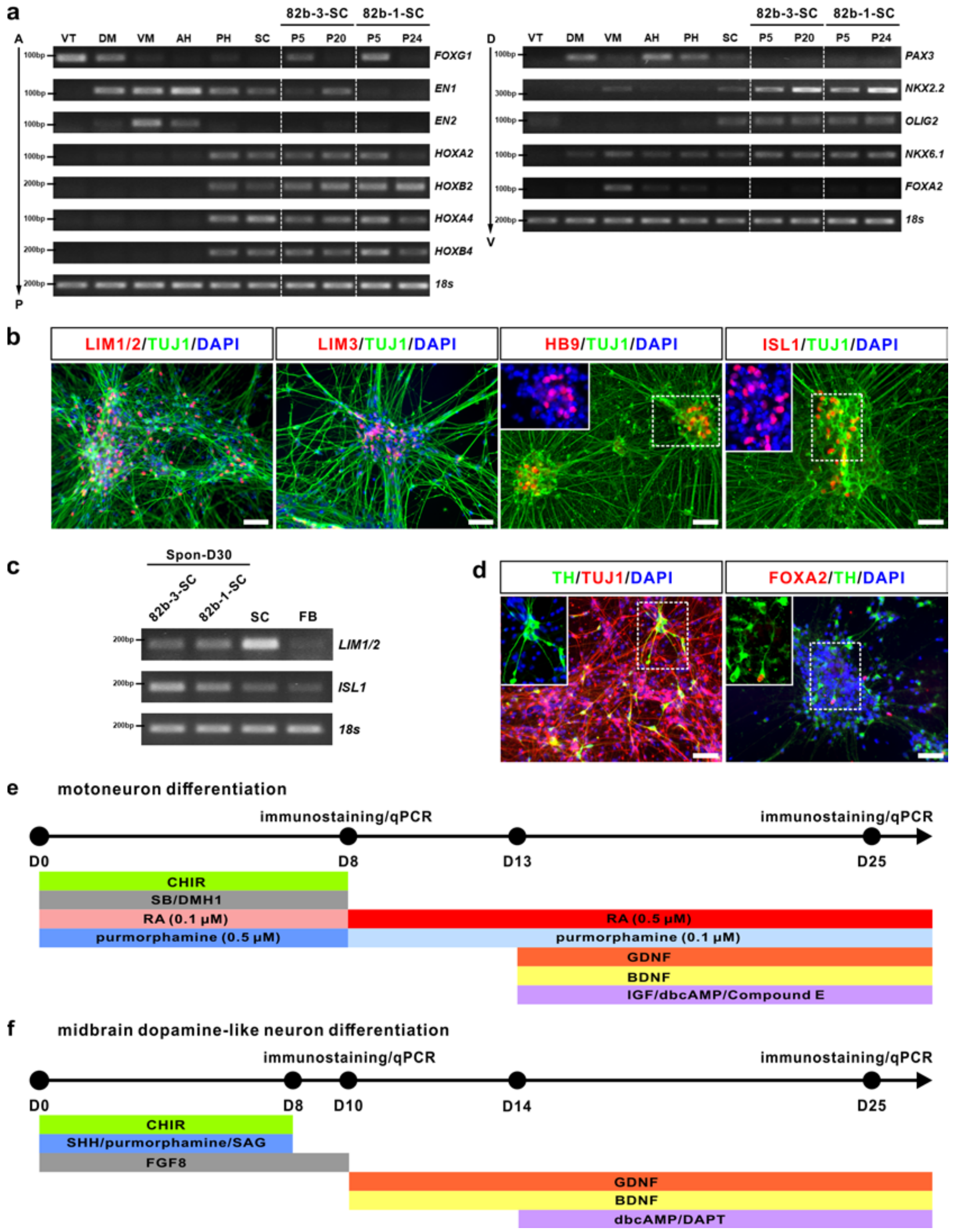

Supplementary Figure 7. iNSCs exhibit a posterior ventral identity but are amenable to patterning into distinct neuronal fates. (**a**) Posterior markers such as *HOX* genes and ventral markers such as *NKX2.2*, *NKX6.1*, and *OLIG2* are readily detectable, whereas anterior and dorsal markers such as *FOXG1* and *PAX3* are absent in long-term expanded iNSC cultures. (**b**) Markers compatible with posterior interneurons and motoneurons such as LIM1/2, LIM3, HB9, and ISL1 are observed in spontaneously differentiated iNSC

cultures. (**c**) RT-PCR analysis confirms the expression of *LIM1/2* and *ISL1* in iNSC cultures after 30 days of spontaneous differentiation. (**d**) Upon spontaneous differentiation, iNSCs also generate occasional TH^+ neurons. However, few of the TH^+ cells co-express FOXA2. (**e, f**) Schematic summary of conditions used for the differentiation of iNSCs into motoneurons (e) and midbrain dopamine-like neurons (f). Human fetal brain RNA samples from different regions are used as controls for characterizing the regional identity of iNSCs. A → P: anterior/posterior axis; D → V: dorsal/ventral axis; VT: ventral telencephalon; DM: dorsal midbrain; VM: ventral midbrain; AH: anterior hindbrain; PH: posterior hindbrain; SC: spinal cord; Spon-D30: day 30 of spontaneous differentiation. Scale bars: 50 μm.

Supplementary Figure 8

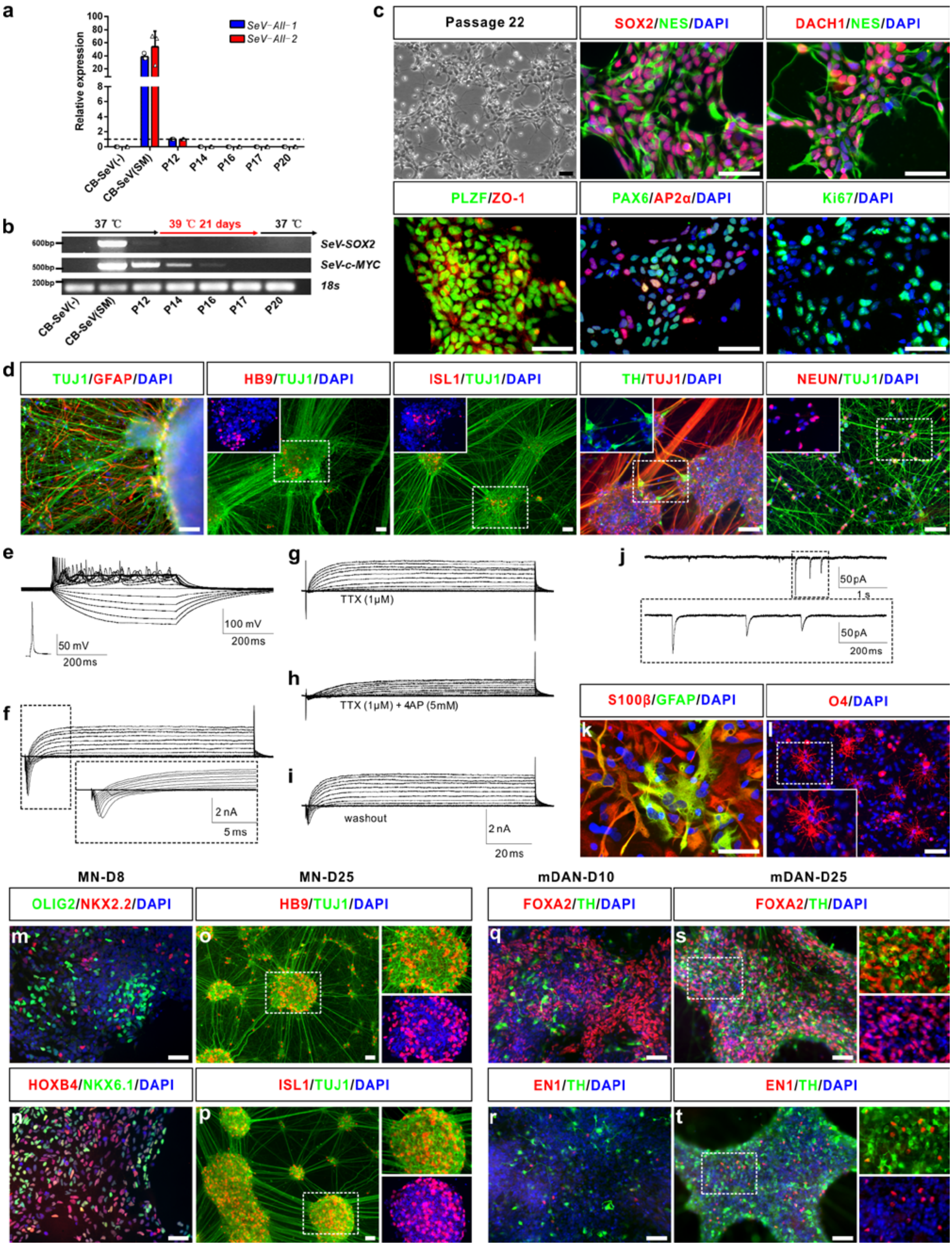

Supplementary Figure 8. Direct conversion of human cord blood CD34^{+} cells (CBCs) into iNSCs. (**a-c**) CB-iNSCs are SeV-free after extended incubation at 39°C and express markers characteristic for early neuroectoderm and neural rosettes (SOX2, NES, DACH1, PLZF, ZO1, and PAX6), neural crest (AP2α, HNK1), and proliferation (Ki67). (**d**) SeV-free CB-iNSCs (passage ⩾ 20) spontaneously differentiate into GFAP^{+} astrocytes and TUJ1^{+} neurons including clusters of HB9^{+}, ISL1^{+} motoneurons and TH^{+} neurons. After extended differentiation, CB-iNSCs (passage 22) give rise to more mature neurons expressing NEUN. (**e-j**) Whole-cell current clamp measurements performed on CB-iNSC-derived neurons reveal the generation of multiple action potentials upon depolarizing current injection (e, n=6). Fast and transient TTX-sensitive inward currents and sustained outward currents are observed in response to depolarizing voltage steps (f-i, n=6). Spontaneous postsynaptic currents are observed in whole-cell voltage-clamp recordings, indicating functional synapse formation in vitro (j, n=6). (**k, l**) CB-iNSCs efficiently differentiate to GFAP^{+}/S100β^{+} astrocytes (k) and, upon subjection to a dedicated protocol[26], O4^{+} oligodendrocytes (l). (**m-p**) Activation of SHH and retinoic acid signaling enables efficient differentiation of CB-iNSCs into OLIG2^{+} and HOXB4^{+} motoneuron progenitors (m, n), which also express NKX2.2 and NKX6.1. Patterned CB-iNSCs can further give rise to an enriched motoneuron population expressing HB9 and ISL1 (o, p). (**q-t**) Inhibition of GSK3β signaling and activation of SHH signaling robustly induce FOXA2 expression in CB-iNSCs (q), which further differentiate into FOXA2^{+}/TH^{+} midbrain dopamine-like neurons (s). EN1^{+}/TH^{+} cells are also observed upon patterning (r, t). Data are presented as mean + s.d.; n = 3. CB-SeV (SM): CBCs infected with SeV-SOX2 and SeV-c-MYC; CB-SeV (-): CBCs without infection. Scale bars: 50 μm.

Supplementary Figure 9

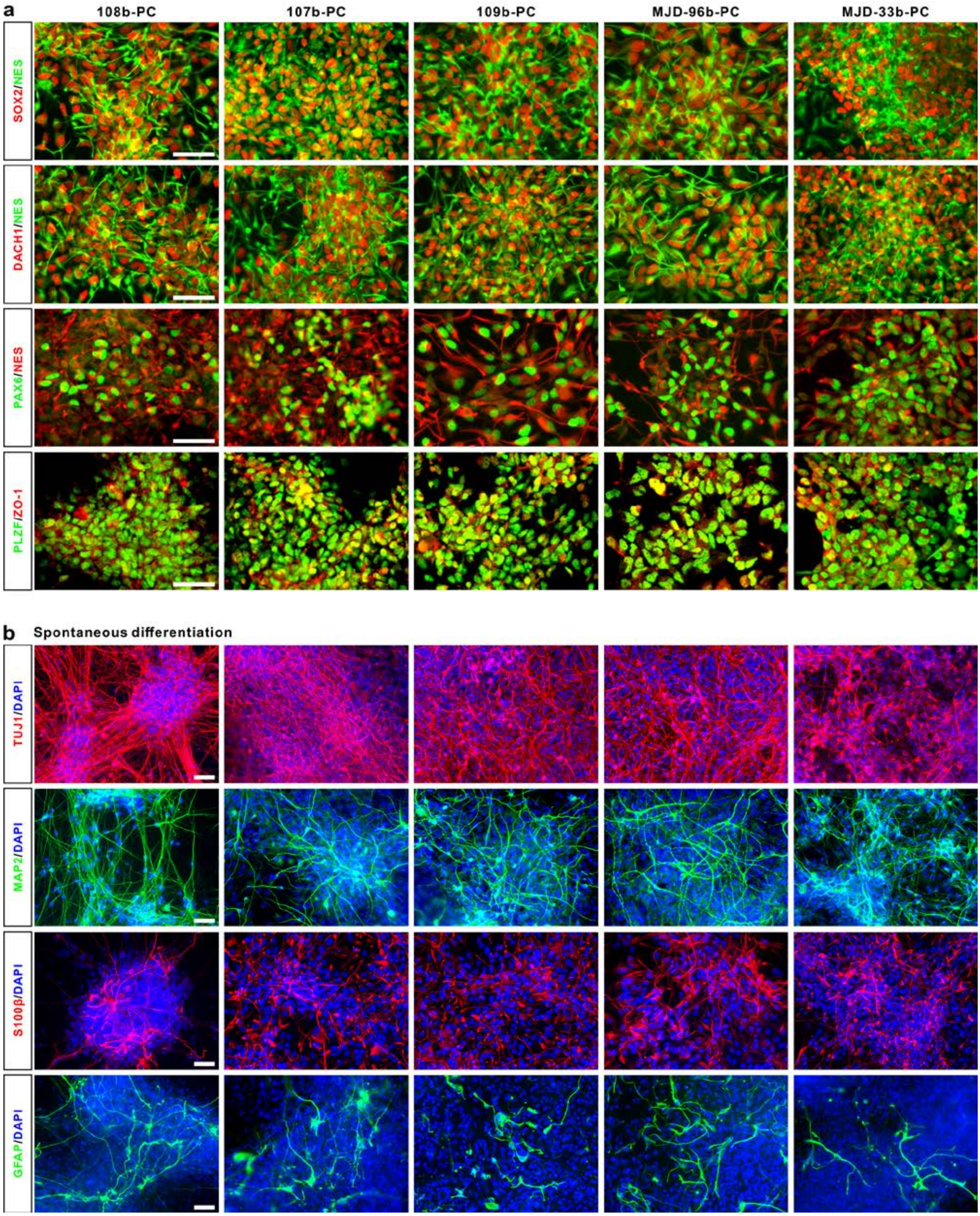

Supplementary Figure 9. Characterization of poly-colony iNSCs generated from individuals of different ages. (**a**) Poly-colony iNSCs generated from 5 individuals are stably self-renewing and express characteristic early neuroectoderm and neural rosette markers, such as SOX2, NES, DACH1, PAX6, PLZF, and ZO1. (**b**) Upon spontaneous differentiation induced by growth factor withdrawal, poly-colony iNSCs give rise to neurons (TUJ1, MAP2) and astroglia (S100β, GFAP). Scale bars: 50 μm.

Supplementary Figure 10

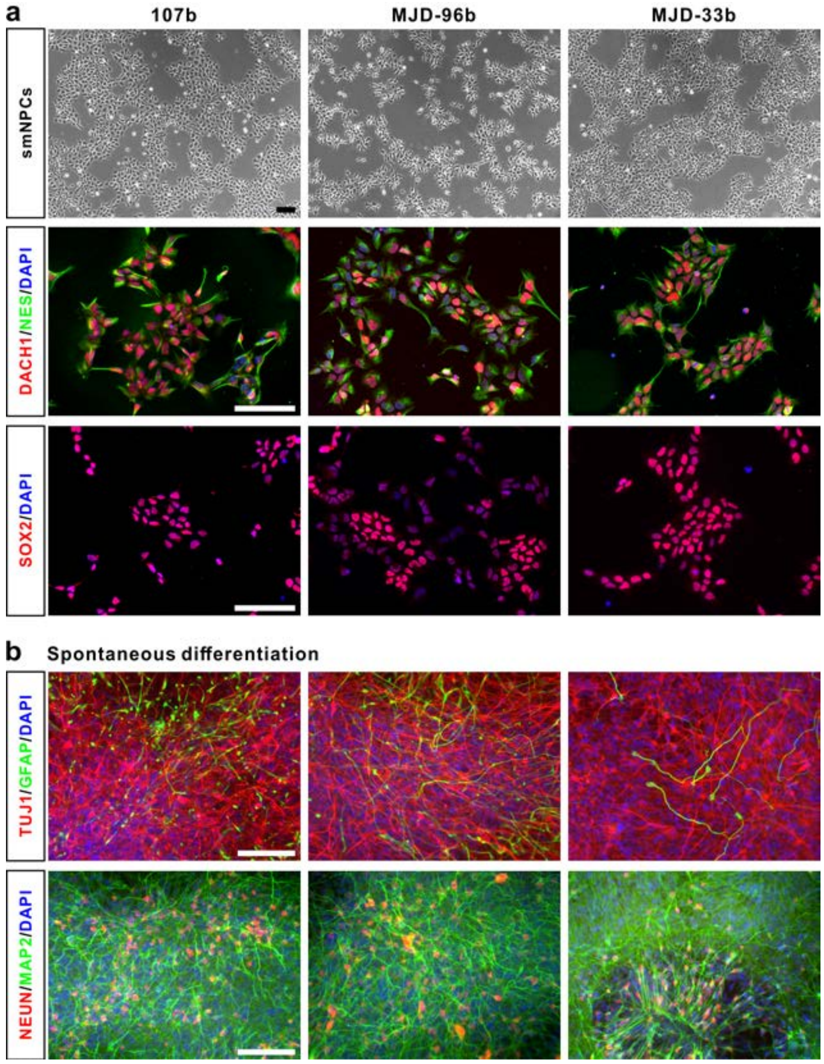

Supplementary Figure 10. Characterization of isogenic PB-iPSC-derived smNPCs. (**a**) smNPCs generated from 3 isogenic PB-iPSCs are stably self-renewing and express characteristic NPC markers, such as SOX2, NES, and DACH1. (**b**) Upon spontaneous differentiation induced by growth factor withdrawal, smNPCs give rise to neurons (TUJ1, MAP2, NEUN) and astroglia (GFAP). Scale bars: 100 µm.

Supplementary Figure 11

a

Correlation between DNAm ages and chronological ages of PBCs

	Horvath	Hannum	99CpGs
Mean absolute deviation (years, n=6)	3.96	15.37	11.15

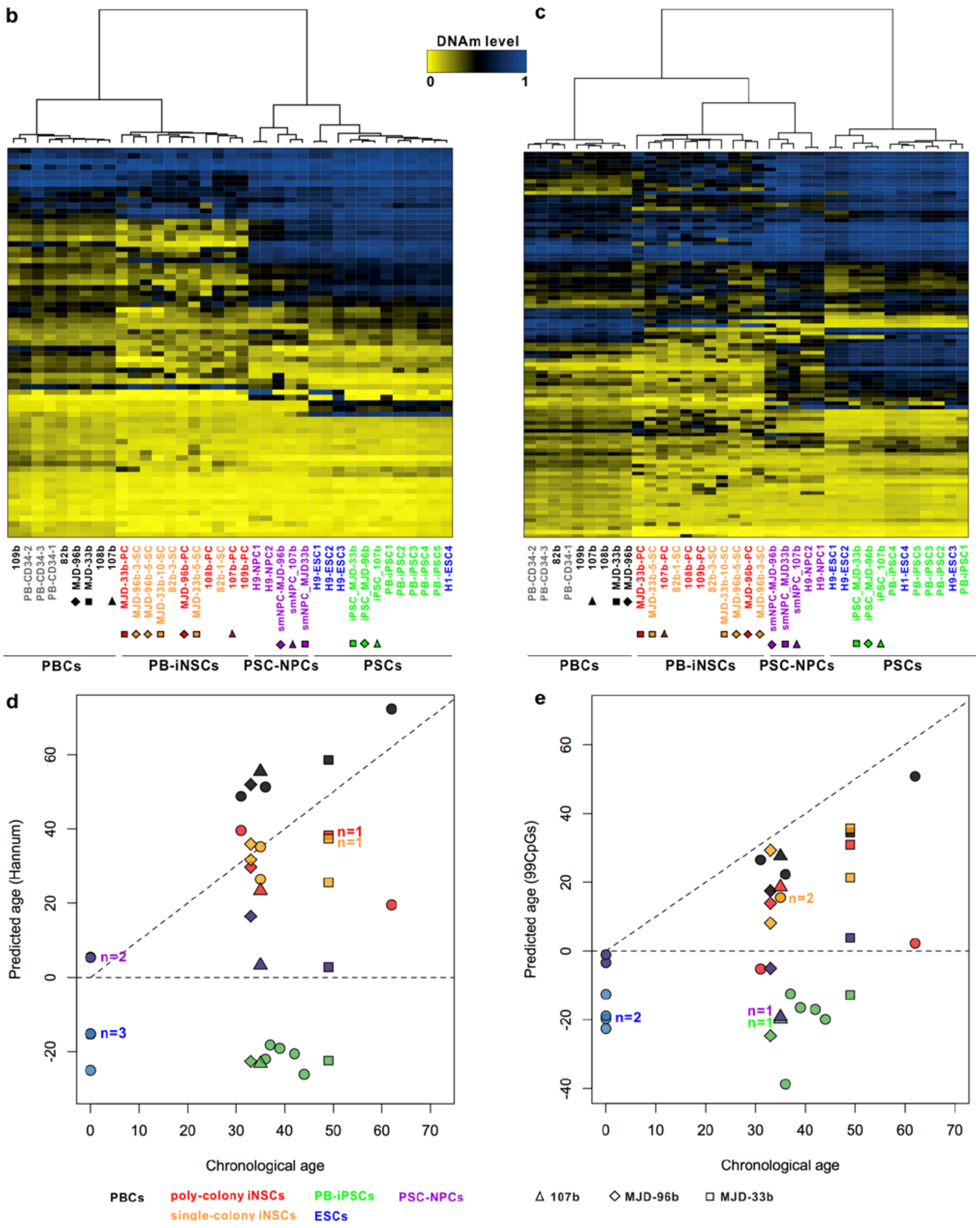

Supplementary Figure 11. Blood-derived iNSCs lose age-associated epigenetic signatures. (**a**) Mean absolute deviation values in three aging models indicate that the Horvath model has superior precision in age prediction in our experimental system. (**b, c**) Heatmap representations of DNAm levels at age-associated CpG sites derived from the Hannum (b) and 99CpGs (c) model[35,36]. In both models, iNSCs show unique age-associated DNAm patterns distinct from PSCs. (**d, e**) Age prediction results from the Hannum (d) and 99CpGs (e) model. Although the DNAm ages predicted by the different models differ, both the Hannum and 99CpGs model recapitulate the pattern depicted in the Horvath model (Fig. 5b), where the DNAm ages of low passage iNSCs lie between those of the parental PBCs and isogenic iPSCs.

Supplementary Figure 12

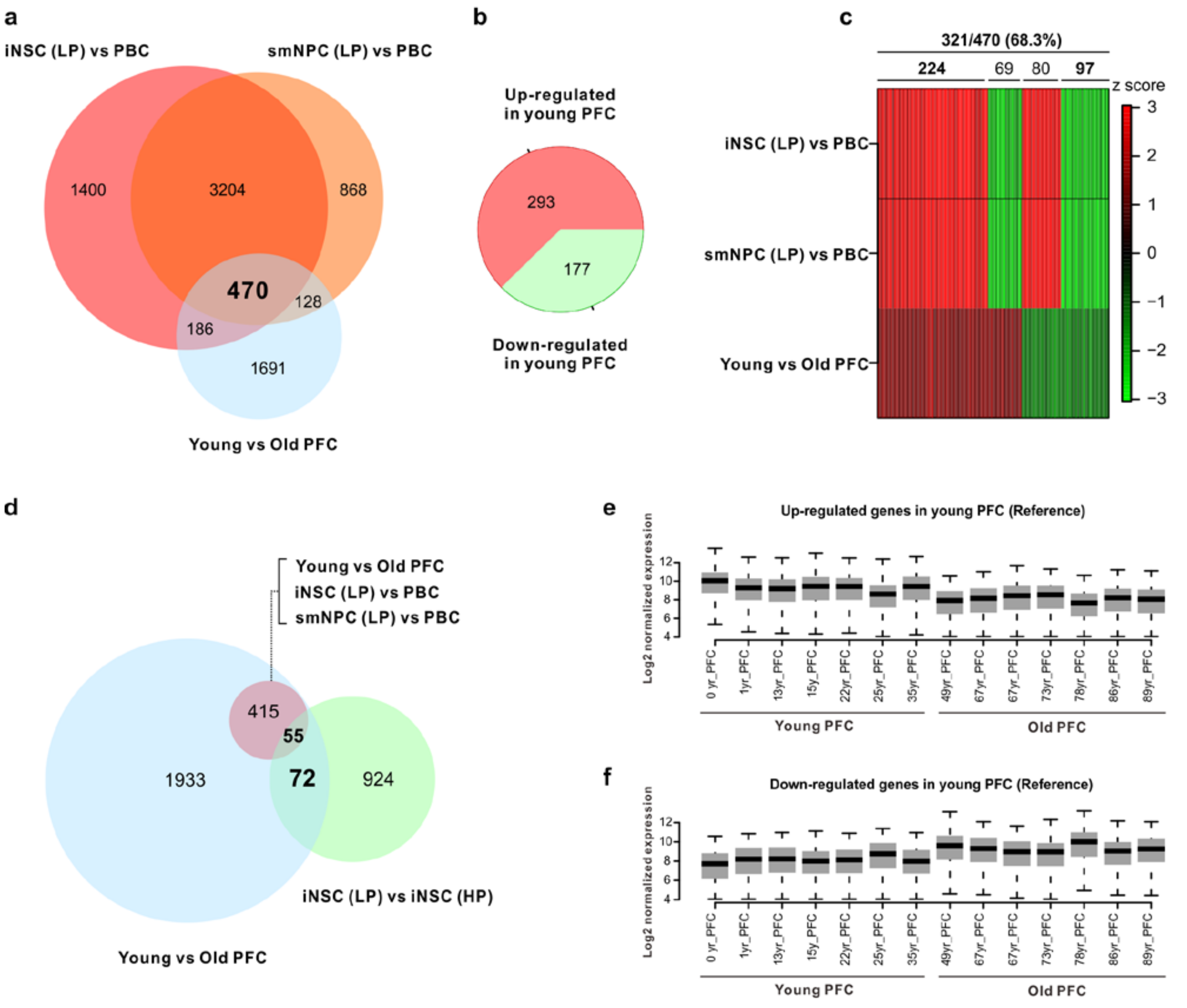

Supplementary Figure 12. RNA seq analysis of PBCs, iNSCs, and smNPCs. (**a**) Number of age-associated genes (based on genes differentially expressed in young (< 49 years of age) and old (⩾ 49 years of age) pre-frontal cortex) that are differentially expressed between low passage iNSCs and donor PBCs (656 genes, 26.5% of all age-associated genes) as well as low passage smNPCs and PBCs (598 genes, 24.2% of all age-associated genes). Overlap analysis shows that iNSCs and smNPCs share the majority of age-associated gene expression changes (470 genes) in comparison to PBCs. (**b**) Within the 470 genes, there are 293 and 177 genes that are up- and down-regulated in the young PFC samples in comparison to the old PFC samples, respectively. (**c**) The majority of age-associated gene expression

alterations in low passage iNSCs and smNPCs vs. their parental PBCs follows the direction of expression change (up or down-regulation) observed in young vs. old PFC tissues (321 out of 470 genes; 68.3%). Specifically, 224 and 97 genes were concordantly up- and down-regulated, respectively, in low passage iNSCs and smNPCs vs. their parental PBCs and in young vs. old PFC. (**d**) Only 127 of the 2,475 age-associated DEGs (55 of the 470 genes) overlap with genes differentially expressed in low vs. high passage iNSCs (127 out of 1051 genes; 12.1%). (**e, f**) Expression level distribution of the 470 genes that are differentially expressed in young vs. old PFC samples as well as in low passage iNSCs and smNPCs vs. PBCs shows a clear separation between the young and old PFC groups. Data are shown for genes with higher expression levels in young (e) and higher expression levels in old (f) PFC samples. Box plots display the median (centre line), the 25th and 75th percentile (upper and lower box, respectively) and 1.5-times the interquartile range from the box (whiskers). Data points outside this region are not shown.

Supplementary Figure 13

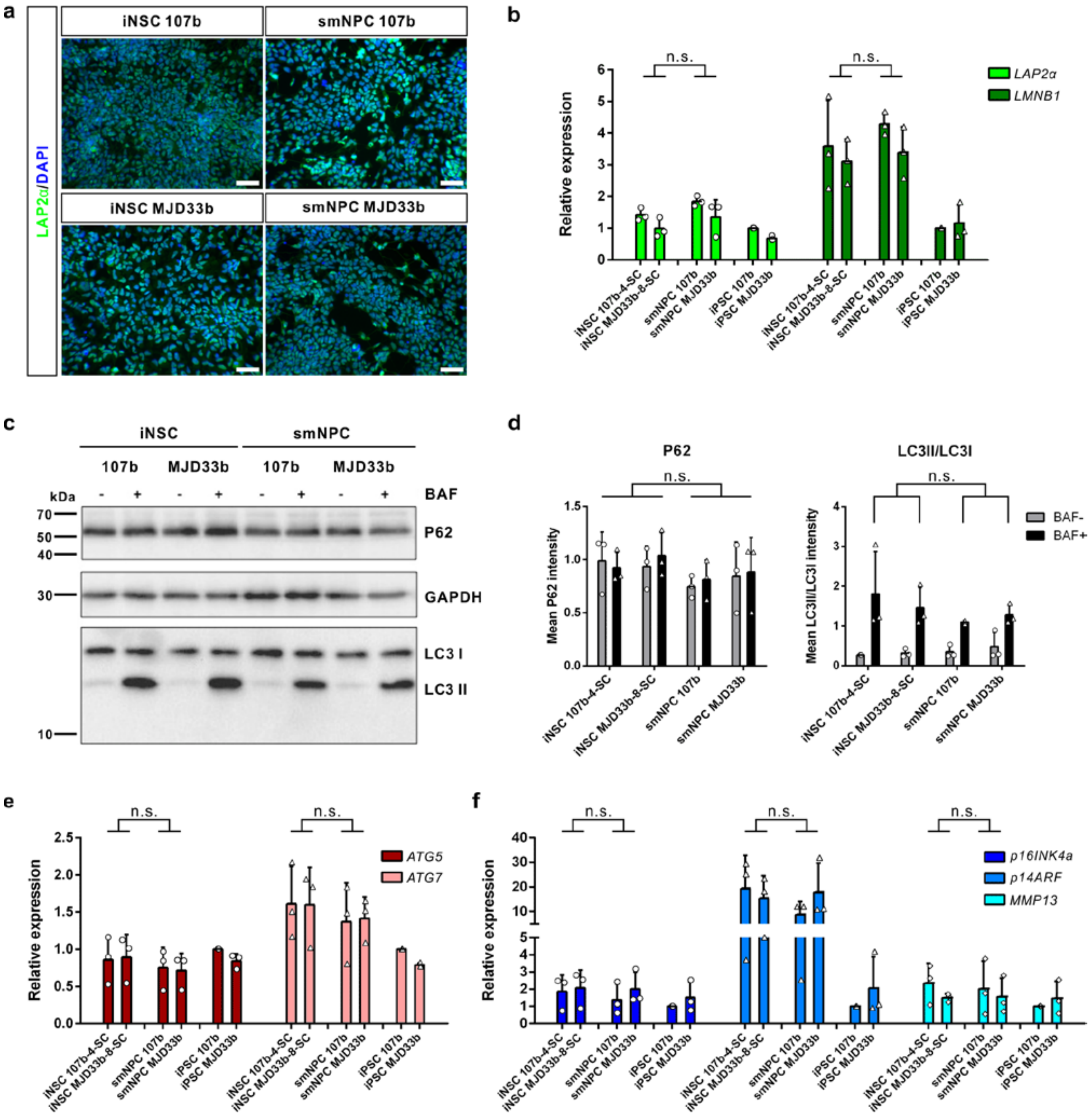

Supplementary Figure 13. Cellular aging hallmarks in iNSCs and isogenic smNPCs. (**a**) Immunostaining of iNSCs and smNPCs shows no overt difference in LAP2α expression. (**b**) qPCR reveals that iNSCs and smNPCs express *LAP2α* and *LMNB1* at similar levels. (**c, d**) Isogenic iNSCs and smNPCs were treated with bafilomycin A (BAF) and analyzed for expression of the autophagy substrate p62 and LC3 forms LC3-I and LC3-II. Quantification of p62 Western blot results (n=3) revealed no significant difference between iNSCs and smNPCs, irrespective of BAF treatment. The autophagic flux during the BAF treatment, evaluated by the

conversion of LC3-I to LC3-II, too, shows no significant difference between iNSCs and smNPCs. (**e**) The autophagy-associated genes *ATG5* and *ATG7* are not differentially expressed in iNSCs and smNPCs. (**f**) Senescence- and apoptosis-associated genes *p16INK4a*, *p14ARF*, and *MMP13* are expressed at comparable levels in iNSCs and isogenic smNPCs. mRNA expression levels of different genes are normalized to iPSC_107b. Data are represented as mean + s.d.; n=3. n.s.: no statistically significant difference after performing Wilcoxon signed rank test or Kruskal Wallis non-parametric ANOVA to compare two or more groups, respectively.

Supplementary Figure 14

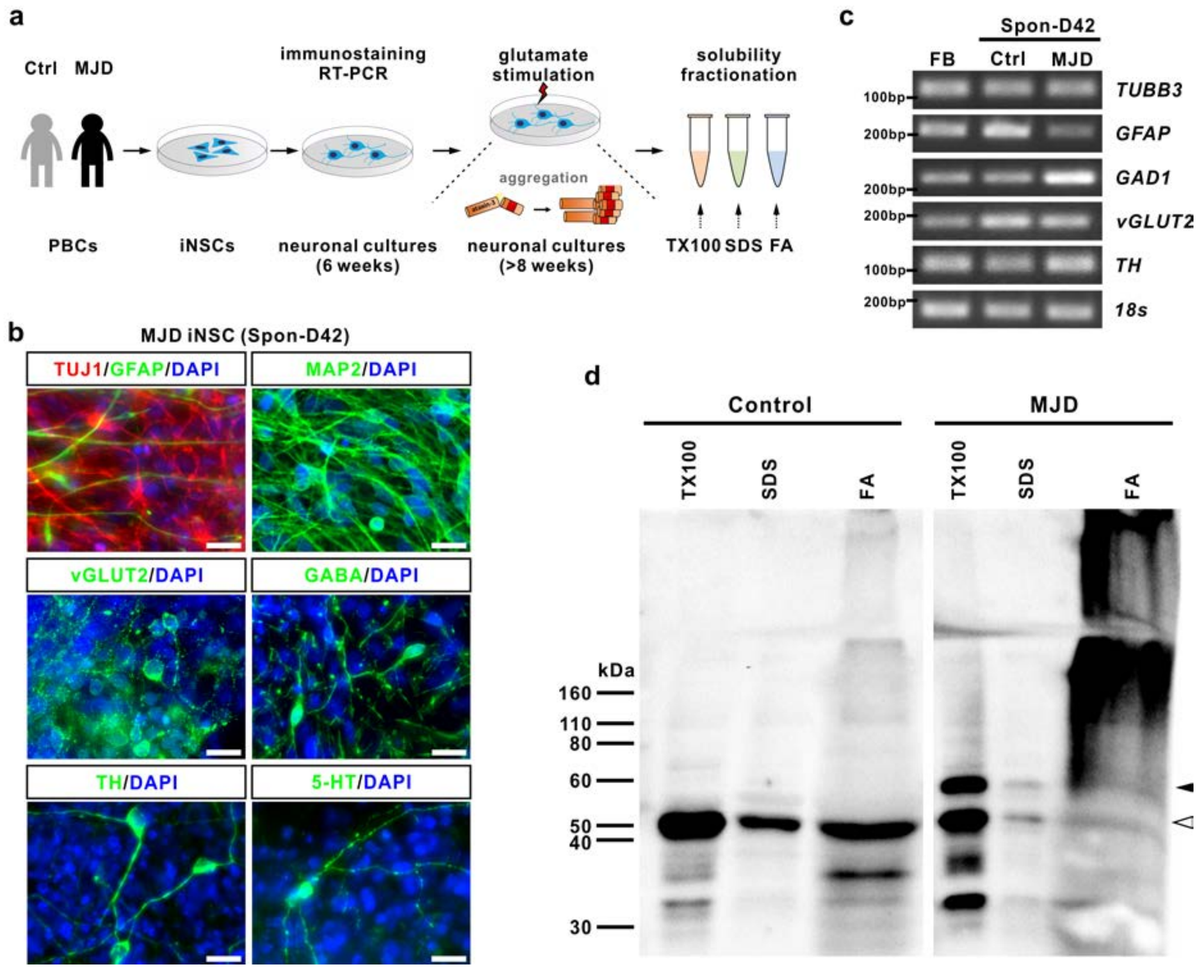

Supplementary Figure 14. Modeling protein aggregation in MJD using iNSC-derived neuronal cultures.

As an exemplar for iNSC-based disease modelling, we chose PB-iNSC-derived neuronal cultures generated from a 49-year-old female MJD patient (iLB-MJD3-33bf, 73/27 CAG repeats, corresponding to patient MJD3 in the study of Koch et al., 2011) and a 35-year-old unrelated healthy female donor. (**a**) Schematic representation of the approach used to assess ataxin-3 aggregation in MJD and control iNSC-derived neuronal cultures. (**b**) iNSCs, differentiated for 6 weeks, give rise to TUJ1- and MAP2-positive neurons as well as GFAP-positive astrocytes. In addition to vGLUT2-positive glutamatergic neurons, the cultures contain GABAergic (GABA), catecholaminergic (TH), and serotonergic (5-HT) neurons. (**c**) RT-PCR analysis confirming expression of *TUBB3*, *GFAP*, *GAD1*, *vGLUT2*, and *TH* in spontaneously differentiated control and MJD iNSC cultures. (**d**) Western blot analysis performed after 8 weeks of differentiation reveals a pronounced

accumulation of ataxin-3-positive complexes in the SDS-insoluble fraction (formic acid, FA) in MJD iNSC-derived neuronal cultures as compared to control cells. Black arrowhead: expanded ataxin-3; blank arrowhead: wild-type ataxin-3. Scale bars: 20 µm.

For these experiments, MJD and control iNSCs were first cultured in poly-L-ornithine (Sigma)/laminin (Life Technologies)-coated plates in N2 medium (DMEM/F12, 1x N2, 1.6 g/l D-glucose (Sigma)) plus 1 µl/ml B27 (with vitamin-A, Life Technologies), 10 ng/ml bFGF and 10 ng/ml EGF (both from R&D) for 2 weeks. Cells were split using trypsin-EDTA and trypsin inhibitor (both from Life Technologies) every 2 to 3 days. Neuronal differentiation was based on a published protocol[44] and was initiated by removing bFGF and EGF from the media and culturing the cells in N2B27 medium supplemented with 300 ng/ml cAMP (Sigma) and 1% Penicillin/Streptomycin. For induction of aggregates, MJD and control iNSC-derived neurons cultured in the absence of bFGF and EGF for 8 weeks as described were washed three times with 2 ml BSS (balanced salt solution) containing (in mM): 25 Tris, 120 NaCl, 15 glucose, 5.4 KCl, 1.8 $CaCl_2$, 0.8 $MgCl_2$, pH 7.4. After treatment with L-glutamate (300 µM, Sigma) in BSS for 30 min, cells were left to recover for 30 min in N2B27 followed by a second 30 min L-glutamate treatment in BSS and subsequently cultured in differentiation media for 18 h. For analysis, cells were washed three times in ice-cold BSS and immediately frozen in liquid N2 until further processing. Initial lysis was performed in RIPA buffer (50 mM Tris, 150 mM NaCl, 0.2% Triton X-100) containing 25 mM EDTA. For analysis of fragmentation and aggregation of ataxin-3 by SDS-PAGE and Western blotting, extracts were analyzed by fractionation for solubility in detergents with increasing strength. Lysates containing 1 – 2 µg/µl total protein dissolved in RIPA buffer including 25 mM EDTA were centrifuged at 16,100 g for 90 min at 4 °C. The pellet fractions were separated from supernatants (Triton X-100-soluble fraction) and homogenized in 300 µl RIPA buffer containing 2% SDS followed by a second centrifugation step at room temperature. The supernatants (SDS-soluble fraction) were removed, and the remaining pellets were incubated for 16 h in 100 % formic acid at 37 °C. After evaporation in a SpeedVac concentrator (Thermo

Scientific) at 45°C, the pellet was dissolved in 4x Laemmli-buffer (SDS-insoluble fraction) followed by pH adjustment with 2 M Tris-base and water was added to a final volume of 60 µl for SDS–PAGE analysis. Gels were loaded with 15 µg of the Triton X-100 fraction, 20 µl of the SDS-soluble fraction and the complete SDS-insoluble fraction. Western blot analyses were performed following standard procedures.

Supplementary Figure 15

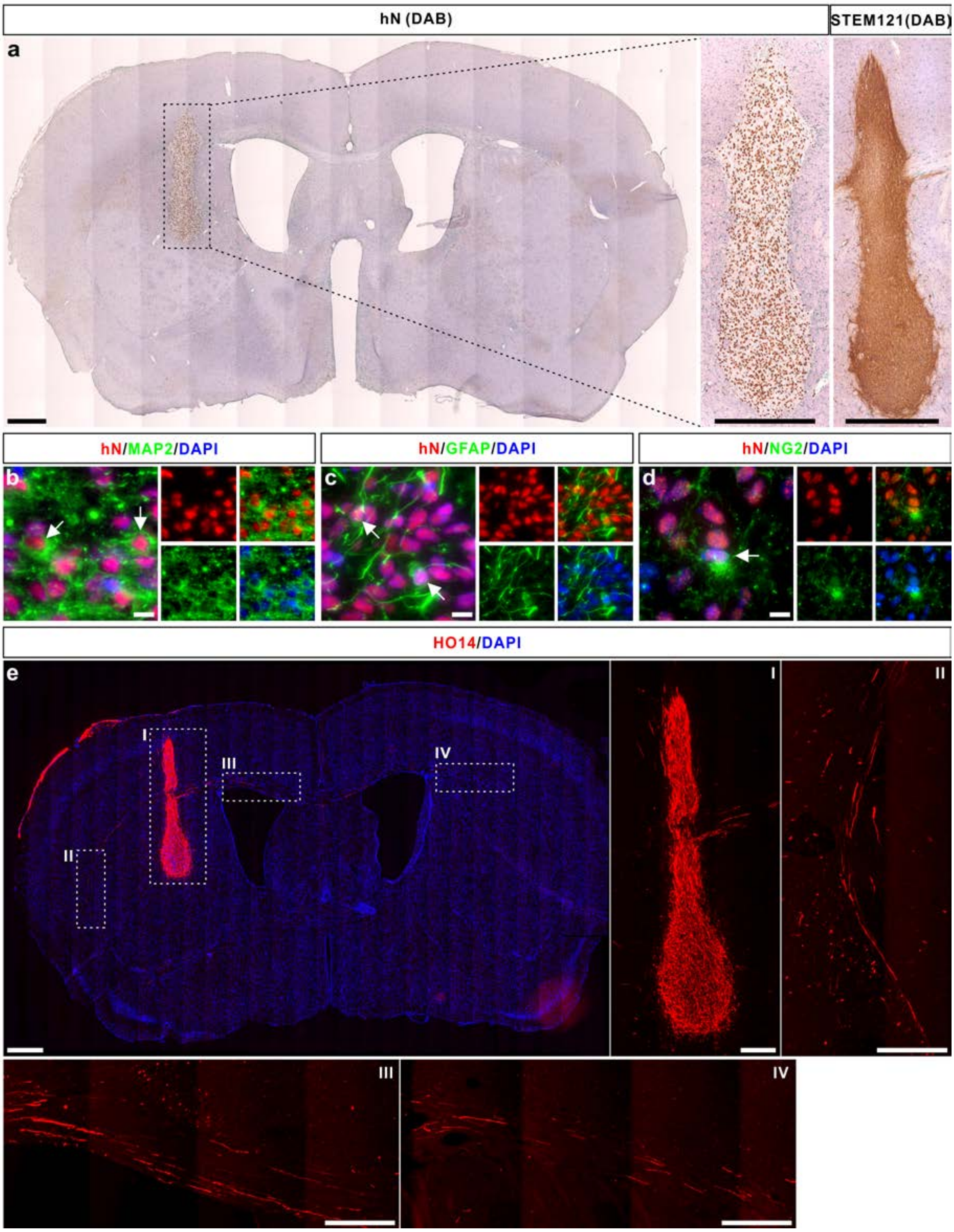

Supplementary Figure 15. Transplantation of iNSCs into adult mouse brain. For these experiments, 100,000 iNSCs were unilaterally injected into the striatum of adult (> 8-week-old) unlesioned Rag2-/- mice. (**a**) Ten to eleven weeks after transplantation, the grafted cells had formed solid grafts delineated by

immunohistochemical staining with antibodies to human nuclei (hN) and the human-specific marker STEM121. (**b**) Immunofluorescence analysis of the transplant with antibodies to MAP2 and human-specific hN shows that iNSCs differentiate into neurons in vivo. (**c**) GFAP expression in hN-positive cells confirms astroglial differentiation of engrafted iNSCs. (**d**) Occasional cells co-expressing NG2 and hN can be found in the transplant, suggesting that iNSCs give rise to oligodendrocytes in vivo. (**e**) Immunofluorescence labeling with the human-specific neurofilament antibody HO14 revealed that engrafted iNSC-derived neurons (I) form long-range axonal projections extending, via the corpus callosum (III), even into the contralateral hemisphere (IV). Scale bars: 500 µm (a, e); 200 µm (e-I, -II, -III, -IV); 10 µm (b-d).

Animal experiments were performed in accordance with the guidelines of the University of Bonn Animal Care Committee, and were approved by the board on proper use of experimental animals (approval number: 84-02.04.2014.A323).

For donor cell preparation, iNSCs were harvested using accutase and concentrated in 0.1% BSA in PBS to 100,000 cells/µl. For transplantation, 1 µl of the cell suspension was injected unilaterally into the striatum. Ten to eleven weeks after transplantation, mice were deeply anesthetized and transcardially perfused with 40 ml PBS followed by 45 ml 4% PFA solution in PBS, pH 7.6. The brains were dissected, post-fixed in 4% PFA solution in PBS, pH 7.6 at 4°C overnight, immersed in 30% sucrose solution at 4°C until they sunk and subjected to cryosectioning. Engrafted cells were identified using immunohistochemical or immunofluorescence staining of coronal sections. For immunofluorescence staining, brain sections were blocked in 10% normal horse serum (NHS), 1% normal goat serum (NGS), and 0.1% Triton X-100 in PBS, followed by incubation with primary and fluorophore-coupled secondary antibodies diluted in 3% NHS, 1% NGS, and 0.1% Triton X-100 in PBS. For histochemistry (a), brain sections were pre-treated with 0.3% H_2O_2 for 15 min, and antigens were visualized using a peroxidase-coupled secondary antibody and a DAB Substrate High Contrast Kit (Zytomed Systems). Images were taken using a ZEISS Observer Z1 microscope.

Supplementary Table 1

Cell populations	Gender	Back-ground	Passage	Clonal state	Chronological age	Age prediction		
						Horvath	Hannum	99CpGs
**PBC_109b**	M	Ctrl			62	62.2	72.3	50.8
**PBC_MJD-33b**	F	MJD			49	41.2	58.6	34.4
**PBC_82b***	F	Ctrl			36	36.0	51.3	22.3
**PBC_107b**	M	Ctrl			35	40.2	55.5	27.6
**PBC_MJD-96b**	M	MJD			33	36.0	51.9	17.5
**PBC_108b**	F	Ctrl			31	38.4	48.8	26.5
**Poly-colony iNSCs**	**Low passage**							
**109b-PC**	M	Ctrl	P5	Poly-colony	62	14.0	19.5	2.2
**MJD-33b-PC**	F	MJD	P4	Poly-colony	49	5.5	38.3	30.9
**107b-PC**	M	Ctrl	P3	Poly-colony	35	7.5	23.4	18.6
**MJD-96b_PC**	M	MJD	P4	Poly-colony	33	13.0	29.7	13.9
**108b-PC**	F	Ctrl	P3	Poly-colony	31	10.8	39.6	-5.2
**Poly-colony iNSCs**	**High passage**							
**MJD-33b-PC**	F	MJD	P18	Poly-colony	49	4.7	43.8	1.8
**107b-PC**	M	Ctrl	P18	Poly-colony	35	3.6	24.5	15.6
**108b-PC**	F	Ctrl	P18	Poly-colony	31	4.4	36.8	18.3
**Single-colony iNSCs**	**Low passage**							
**MJD-33b-5-SC**	F	MJD	P4	Single-colony	49	7.4	37.3	35.6
**MJD-33b-10-SC**	F	MJD	P5	Single-colony	49	6.3	25.6	21.3
**82b-1-SC**	F	Ctrl	P5	Single-colony	35	9.3	26.4	15.5
**82b-3-SC**	F	Ctrl	P5	Single-colony	35	8.0	35.1	15.6
**MJD-96b-3-SC**	M	MJD	P5	Single-colony	33	1.8	31.7	29.3
**MJD-96b-5-SC**	M	MJD	P5	Single-colony	33	11.1	35.9	8.1
**Single-colony iNSCs**	**High passage**							
**MJD-33b-10-SC**	F	MJD	P21	Single-colony	49	5.8	33.0	28.7
**82b-1-SC**	F	Ctrl	P21	Single-colony	35	3.2	38.3	17.2
**82b-3-SC**	F	Ctrl	P21	Single-colony	35	4.8	34.5	40.3
**MJD-96b-5-SC**	M	MJD	P20	Single-colony	33	4.6	28.6	-9.1
**iNSC-Subclones**	**High passage**							
**82b-3-subclone1**	F	Ctrl	P23	Subclone	35	9.8	44.2	30.7
**82b-3-subclone2**	F	Ctrl	P24	Subclone	35	3.5	36.6	20.3
**82b-3-subclone3**	F	Ctrl	P24	Subclone	35	2.4	28.0	14.5
**82b-3-subclone4**	F	Ctrl	P25	Subclone	35	4.7	27.8	13.9
**82b-3-subclone5**	F	Ctrl	P23	Subclone	35	2.3	28.1	17.5
**Differentiated iNSCs**	**High passage, 8 weeks of spontaneous differentiation**							
**MJD-33b-10-SC**	F	MJD	P21	Single-colony	49	8.2	14.0	29.2
**82b-1-SC**	F	Ctrl	P23	Single-colony	35	3.8	26.1	14.1
**82b-3-SC**	F	Ctrl	P23	Single-colony	35	5.5	18.1	35.3
**82b-3_subclone1**	F	Ctrl	P24	Subclone	35	9.5	30.7	28.9
**82b-3_subclone2**	F	Ctrl	P25	Subclone	35	5.8	23.7	19.8
**82b-3_subclone3**	F	Ctrl	P26	Subclone	35	3.2	18.9	12.3
**107b-PC**	M	Ctrl	P20	Poly-colony	35	3.5	12.2	12.2
**MJD-96b-5-SC**	M	MJD	P21	Single-colony	33	3.5	5.6	-8.5

Supplementary Table 1. Age prediction of iNSCs and parental PBCs. Age prediction results for PBCs, poly- and single-colony iNSCs at low and high passages, iNSC subclones as well as spontaneously differentiated iNSC cultures according to the Horvath, Hannum, and 99CpGs model[34,35,36]. F: female; M: male; Ctrl: control; MJD: Machado Joseph disease. * PBC_82b (age 36) was re-recruited from the same donor one year after the establishment of iNSC_82b-1-SC and iNSC_82b-3-SC.

Nomenclature of different cell populations:

- PBCs:

  "PBC_**109b**", "**109b**"→**recruitment code**

- Poly-colony iNSCs:

  "iNSC_**109b**-**PC**", "**109b**"→**recruitment code**, "**PC**"→**poly-colony origin**

- Single-colony iNSCs:

  "iNSC_**82b**-3-**SC**", "**82b**"→**recruitment code**, "3"→colony number, "**SC**"→**single-colony origin**

- iNSC subclones:

  "iNSC_**82b**-**3**-**subclone1**", **82b**"→**recruitment code**, "3"→colony number, "**subclone1**"→**subclone number**

Supplementary Table 2

Cell populations	Gender	Back-ground	Passage	Clonal state	Chronological age			
						Horvath	Hannum	99CpGs
**Isogenic PB-iPSCs**								
**PB-iPSC_MJD-33b**	F	MJD	P25	Poly-colony	49	-0.1	-22.4	-12.8
**PB-iPSC_107b**	M	WT	P23	Poly-colony	35	-0.3	-23.2	-19.9
**PB-iPSC_MJD-96b**	M	MJD	P25	Poly-colony	33	-0.5	-22.6	-24.7
**PB-iPSCs (GSE40790)**								
**PB-iPSC1**					44	-0.2	-26.1	-19.9
**PB-iPSC3**					42	-0.3	-20.6	-17.0
**PB-iPSC2**					39	-0.1	-19.1	-16.5
**PB-iPSC5**					37	-0.4	-18.2	-12.5
**PB-iPSC4**					36	-0.6	-22.0	-38.7
**ESCs (GSE38216)**								
**H9-ESC1**					0	-0.1	-15.2	-18.9
**H9-ESC2**					0	-0.2	-15.2	-19.8
**H9-ESC3**					0	-0.1	-25.0	-22.6
**H1-ESC4**					0	-0.4	-15.3	-12.6
**iPSC-smNPCs**								
**smNPC_MJD-33b**	F	MJD	P6		49	0.7	2.7	3.8
**smNPC_107b**	M	WT	P6		35	0.3	3.3	-19.2
**smNPC_MJD-96b**	M	MJD	P6		33	1.3	16.4	-5.1
**smNPC_MJD-33b**	F	MJD	P18		49	4.2	15.2	12.7
**smNPC_107b**	M	WT	P18		35	2.3	9.4	-9.8
**smNPC_MJD-96b**	M	MJD	P18		33	1.7	24.4	6.9
**ESC-NPCs (GSE38216)**								
**H9-ESC-NPC1**					0	1.8	5.3	-1.1
**H9-ESC-NPC2**					0	2.0	5.5	-3.4
**Differentiated smNPCs**	**High passage, 8 weeks of spontaneous differentiation**							
**smNPC_MJD-33b**	F	MJD	P18		49	4.0	3.9	10.7
**smNPC_107b**	M	WT	P18		35	4.2	3.5	-9.5
**smNPC_MJD-96b**	M	MJD	P18		33	1.7	11.9	18.5

Supplementary Table 2. Age prediction of PSCs and PSC-derived neural cells. Age prediction results for PB-iPSCs, ESCs, isogenic iPSC-derived smNPCs, ESC-derived NPCs as well as spontaneously differentiated smNPC cultures according to the Horvath, Hannum, and 99CpGs model[34,35,36]. PB-iPSC1-5: iPSCs generated from the PB-CD34$^+$ cells[33]. The accession numbers of publicly available data sets used in this study are GSE40790 (PB-iPSCs)[33] and GSE38216 (ESCs and ESC-NPCs)[32].

Supplementary Table 3. Antibodies used in this study

Primary antibody	Host	Dilution	Supplier
AP2α	Mouse	1:300	DSHB
Ataxin-3	Mouse	1:1000	Millipore
BRN3A	Rabbit	1:500	Millipore
DACH1	Rabbit	1:100	Protein Tech
EN1	Goat	1:50	Santa Cruz
FOXA2	Goat	1:100	R&D
GABA	Rabbit	1:500	Sigma
GAPDH	Mouse	1:1000	Santa Cruz
GFAP	Rabbit	1:500	Millipore
vGLUT2	Goat	1:300	Abcam
vGLUT2	Mouse	1:300	Abcam
HB9	Mouse	1:300	DSHB
HNK1	Mouse	1:300	Sigma
HO14	Rat	1:50	Gift from Dr. Virginia Lee
HOXB4	Rat	1:300	DSHB
Human nuclei	Mouse	1:100	Millipore
ISL1	Mouse	1:300	DSHB
Ki67	Mouse	1:100	Dako
LAP2α	Rabbit	1:500	Abcam
LC3B	Mouse	1:200	ENZO
LIM1/2 (supernatant)	Mouse	1:5	DSHB
LIM3 (supernatant)	Mouse	1:10	DSHB
LMX1A	Rabbit	1:800	Millipore
MAP2	Rabbit	1:500	Millipore
MBP	Rat	1:200	Abcam
NES	Mouse	1:200	R&D
NES	Rabbit	1:200	Novus Biologicals
NEUN	Mouse	1:100	Millipore
NG2	Rabbit	1:200	Millipore
NKX2.2	Mouse	1:300	DSHB
NKX6.1	Mouse	1:300	DSHB
O4	Mouse	1:100	R&D
OLIG2	Rabbit	1:200	Millipore
P62	Mouse	1:1000	Abnova
PAX6	Rabbit	1:300	Covance
PERIPHERIN	Goat	1:250	Santa Cruz
PLZF	Mouse	1:50	Calbiochem
S100β	Mouse	1:1000	Sigma
SEROTONIN (5-HT)	Rabbit	1:500	Sigma
SOX2	Mouse	1:100	R&D
SOX10	Goat	1:100	Santa Cruz
STEM121	Mouse	1:1000	Stem Cells
TH	Rabbit	1:1000	Millipore
TUJ1	Mouse	1:1000	Covance

TUJ1	Rabbit	1:1000	Covance
TUJ1	Chicken	1:500	Millipore
ZO-1	Rabbit	1:100	Life Technologies

Secondary antibody	Dilution	Supplier
Alexa 488-goat anti mouse IgG	1:1000	Life Technologies
Alexa 488-goat anti rabbit IgG	1:1000	Life Technologies
Alexa 555-donkey anti goat IgG	1:1000	Life Technologies
Alexa 555-goat anti mouse IgG	1:1000	Life Technologies
Alexa 555-goat anti mouse IgM	1:1000	Life Technologies
Alexa 555-goat anti rabbit IgG	1:1000	Life Technologies
Alexa 555-goat anti rat IgG	1:1000	Life Technologies
Alexa 647-goat anti chicken IgG	1:500	Life Technologies
HRP-horse anti mouse IgG	1:1000	Cell Signaling Technology
Cy3-Streptavidin	1:200	Vector Labs

Supplementary Table 4. PCR Primers used in this study

Gene	Forward	Reverse
ATG5	AGAAGCTGTTTCGTCCTGTGG	AGGTGTTTCCAACATTGGCTC
ATG7	ATGATCCCTGTAACTTAGCCCA	CACGGAAGCAAACAACTTCAAC
CD117	GAACACGCACCTGCTGAAAT	ATTGATCCGCACAGAATGGT
CD71	GATTTCGGGAATGCTGAGAA	GCACCGTTATTTTGTTTACGC
CDH2	TTCGGGTAATCCTCCCAAATC	CCACAATCCTGTCCACATCT
EN1	CGTGGCTTACTCCCCATTTA	TCTCGCTGTCTCTCCCTCTC
EN2	CCTCCTGCTCCTCCTTTCTT	GACGCAGACGATGTATGCAC
FOXA2	ACACCACTACGCCTTCAACC	GCCTTGAGGTCCATTTTGTG
FOXG1	TGGGAGATAGGAAAGAGGTGAAAA	GCACCAGGCTGTTGATGCT
GAD1	GCTGGGGCTGCACTTGGCTT	CACCTCCCCAGGCAGCATCG
GFAP	ATCGAGAAGGTTCGCTTCCT	CAGCCTCAGGTTGGTTTCAT
vGLUT2	TCAGATTCCGGGAGGCTACA	TGGGTAGGTCACACCCTCAA
HOXA2	GCTCGCTGAGTGCCTGACAT	GGAGGAGGAATCAGTGTCGAGTG
HOXB2	GCCGACTCCTGTCTCCAGCTAT	ACTGCAGGTCGATGGCACAG
HOXA4	CAGCAGGTCTTGGAGCTGGA	TCTTGACCTGGCGCTCAGAC
HOXB4	CTACTGCCGCTGCTGGAAGA	TGTGTGTGTGTTACCGTGACCAA
LAP2α	GCAGGCAGACATTAGTCAAGC	CGACCTACAGTGGCATTTCC
LMNB1	GAGGTTGCTCAAAGAAGTACAGTC	TTACATAATTGCACAGCTTCTATTG
MMP13	GTCATGCCAGCAAATTCCA	TCCAGCCACGCATAGTCATA
NES	GGAGAAGGACCAAGAACTG	ACCTCCTCTGTGGCATTC
NG2	ACTTGCATCCGCGGCTTCCTTCTT	ACAACGTGGCCCAGCCCTCTA
NKX2.2	TGCCTCTCCTTCTGAACCTTGG	GCGAAATCTGCCACCAGTTG
NKX6.1	GCCTCGGAGAACGAGGAAGA	CGCTGCTGGACTTGTGCTTC
OCT4	GTGGAGGAAGCTGACAACAA	ATTCTCCAGGTTGCCTCTCA
OLIG2	GCTGGCGCGCAACTACATCC	AAGCCAGCGTGGTGGCCC
P14ARF	TCTTGGTGACCCTCCGGATT	CGGGATGTGAACCACGAAAAC
P16INK4a	GGGTCGGGTAGAGGAGGTG	ACCGTAACTATTCGGTGCGT
PAX3	TCCATACGTCCTGGTGCCAT	TTCTCCACGTCAGGCGTTG
PAX6	AATAACCTGCCTATGCAACCC	AACTTGAACTGGAACTGACACAC
PDGFRα	CTATCCACACTGTCAAACAGGTTG	ACTGCTGGACTGAGAAGTTTCATC
PLP	CTGCTCACCTTCATGATTGC	TGACTTGCAGTTGGGAAGTC
SALL4	GCGAGCTTTTACCACCAAAG	CACAACAGGGTCCACATTCA
SeV-All-1	GGATCACTAGTGATATCGAGC	ACCAGACAAGAGTTTAAGAGATATGTATC
SeV-All-2	TGGCTAAGAACATCGGAAGG	GTTTTGCAACCAAGCACTCA
SeV-SOX2	ACAAGAGAAAAAACATGTATGG	ATGCGCTGGTTCACGCCCGCGCCCAGG
endogenous SOX2	GTATCAGGAGTTGTCAAGGCAGAG	TCCTAGTCTTAAAGAGGCAGCAAAC
SeV-c-MYC	TAACTGACTAGCAGGCTTGTCG	TCCACATACAGTCCTGGATGATGATG
TH	ACTGGTTCACGGTGGAGTTC	TCTCAGGCTCCTCAGACAGG
TUBB3	CCATCTTGCTGCCGACAC	CAATAAGACAGAGACAGGAG
18s	TTCCTTGGACCGGCGCAAG	GCCGCATCGCCGGTCGG
